# Phosphorylation of USP33 by CDK1 stabilizes the mTORC2 component SIN1

**DOI:** 10.1038/s41419-025-07869-6

**Published:** 2025-07-22

**Authors:** Yalei Wen, Caishi Zhang, Mingchao Liang, Xiao Yang, Hu Zeng, Rui Wan, Xiuqing Ma, Lei Huang, Mei Li, Qiushi Zhang, Liheng Li, Shengying Qin, Tongzheng Liu

**Affiliations:** 1 Research Institute for Maternal and Child Health, The Affiliated Guangdong Second Provincial General Hospital, Postdoctoral Research Station of Traditional Chinese Medicine, School of Pharmacy, Jinan University, Guangzhou, China; 2 State Key Laboratory of Bioactive Molecules and Druggability Assessment/International Cooperative Laboratory of Traditional Chinese Medicine Modernization and Innovative Drug Development of Ministry of Education (MOE) of China/College of Pharmacy, Jinan University, Guangzhou, China; 3 Jianli Traditional Chinese Medicine Hospital, Jingzhou, China; 4 The Affiliated Shunde Hospital of Jinan University, Foshan, China; 5 The Sixth Affiliated Hospital of Jinan University, Dongguan, China; 6 Department of Interventional Radiology, The Affiliated Guangdong Second Provincial General Hospital of Jinan University, Guangzhou, China; 7 Department of Oncology, The First Affiliated Hospital of Jinan University, Guangzhou, China; 8 Clinical Medical Research Institute, Jinan University, Guangzhou, China

**Keywords:** Oncogenes, Ubiquitylation

## Abstract

Understanding the mechanisms underlying chemoresistance is critical for improving cancer therapies. SIN1 plays a pivotal role in maintaining mTORC2 integrity and activation, which regulates key cellular processes. In this study, we demonstrate that elevated SIN1 expression in pancreatic ductal adenocarcinoma (PDAC) correlates with poor patient survival outcomes. Conversely, SIN1 deletion reduces tumor growth and enhances PDAC sensitivity to chemotherapy. We identify USP33 as a bona fide deubiquitanase of SIN1, essential for its stabilization in PDAC. This stabilization promotes chemoresistance by activating the mTORC2-AKT pathway. Additionally, we show that CDK1 directly phosphorylates USP33, enhancing its deubiquitinase activity toward SIN1 and driving PDAC progression. Inhibition or genetic ablation of CDK1 significantly diminishes these malignant phenotypes. Furthermore, we observe a strong positive correlation between CDK1, USP33, and SIN1 expressions in PDAC tissues. Our results provide compelling preclinical evidence that targeting the CDK1–USP33 axis may offer a promising therapeutic strategy to destabilize SIN1 and overcome chemoresistance in PDAC and potentially other aggressive cancers.

## Introduction

Pancreatic ductal adenocarcinoma (PDAC) remains one of the most lethal cancers worldwide, with a dismal 5-year survival rate of <10% [1]. Gemcitabine, a deoxynucleotide analog, is the standard first-line drug for PDAC, but its efficacy is limited, with a response rate of ~20% and a median survival of only 6 months [2]. However, nearly all patients either fail to respond or rapidly develop resistance to gemcitabine, leading to relapse or metastasis [3]. Therefore, identifying the key mechanisms driving chemoresistance is critical for uncovering novel therapeutic targets that could significantly improve clinical outcomes for PDAC patients.

The mammalian target of rapamycin complex 2 (mTORC2) is primarily composed of mechanistic target of rapamycin kinase (mTOR), stress-activated protein kinase-interacting protein 1 (SIN1), rapamycin-insensitive companion of mTOR (Rictor), mammalian lethal with SEC13 protein 8 (mLST8), and several other components [4]. Among these, SIN1 is an essential subunit by interacts with Rictor, linking Rictor to mLST8, and recruiting mTORC2 substrates, making it crucial for mTORC2 assembly and activation [5]. In response to various growth signals and stress stimuli, mTORC2 is activated and phosphorylates a range of substrates, including protein kinase B (AKT), protein kinase C (PKC), and serum and glucocorticoid-regulated kinase (SGK), thereby critically regulating diverse cellular processes such as cell proliferation, apoptosis, and cell metabolism [6]. Notably, PDK1 phosphorylates AKT at Thr308 in the catalytic domain, which primes AKT for subsequent phosphorylation at Ser473 in the hydrophobic motif by mTORC2, thus leading to the full activation of AKT [7]. Activated AKT then phosphorylates a large number of apoptotic factors, transcription factors, or oncogenic factors such as Forkhead Box O (FOXO), glycogen synthase kinase 3β (GSK3β), and cyclin-dependent kinase inhibitor 27 (p27), which critically control cell proliferation and apoptosis [8]. The mTORC2-AKT signaling pathway is frequently dysregulated in various cancers, including PDAC [9]. For instance, SIN1 is significantly upregulated in breast cancer, colorectal cancer, and hepatocellular carcinoma, leading to sustained mTORC2 and AKT activation and contributing to chemoresistance [10–12]. Therefore, targeting SIN1 to modulate deregulated mTORC2 signaling represents a promising therapeutic strategy for treating cancers and overcoming chemoresistance.

The mechanism underlying the aberrant upregulation of SIN1 in human cancers remains poorly understood. In this study, we identify ubiquitin-specific protease 33 (USP33) as a key deubiquitinating enzyme (DUB) of SIN1, promoting SIN1 stability and SIN1-driven tumor progression in PDAC. Unexpectedly, we find that cyclin-dependent kinase 1 (CDK1) directly activates USP33 to deubiquitinate SIN1, while targeting CDK1 significantly sensitizes PDAC cells to chemotherapy in a SIN1-dependent manner. Overall, our study reveals that the CDK1–USP33–SIN1 axis is a critical regulator of chemoresistance, suggesting novel therapeutic targets for treating PDAC and potentially other aggressive cancers with upregulated SIN1.

## Materials and methods

### Cell culture, plasmids, and antibodies

HEK293T, PANC-1, MIA PaCa-2 cells, and other lines were obtained from American Type Culture Collection (ATCC). HEK293T, PANC-1 cells were cultured in Dulbecco’s modified Eagles’s medium (Gibco) supplemented with 10% fetal bovine serum (FBS) (Gibco). MIA PaCa-2 cells were cultured in Dulbecco’s modified Eagles’s medium (Gibco) supplemented with 10% FBS and 2.5% horse serum (Gibco). All cell lines were mycoplasma-free and authenticated by short tandem repeat DNA profiling analysis.

USP33, SIN1 and CDK1 were cloned into pIRES-Flag-S, pLV3-Flag-S, pLV3-Flag, pLV5-HA-S, pLVX3-GST, PET28A-His and pGEX4T-1-GST vectors, respectively. All site mutants of USP33, SIN1 were generated by site-directed mutagenesis and identified by sequencing. In shRNA experiments, pLKO.1-scramble shRNA was used as a negative control with the sequence of CCTAAGGTTAAGTCGCCCTCG. CDK1 shRNA was gifted from Dr. Bo Yang (Zhejiang University, China). The sequence for homo sapiens shCDK1 is 5’-CTGTACTTCGTCTTCTAATT A-3’. The sequences for homo sapiens shUSP33 #1 and #2 are 5’-GTGGAATTTGTCAGCAGATAT-3’ and 5’-GCAACAGTGATAGAGCAGAAA-3’. The sequences for homo sapiens shSIN1#1 and #2 are 5’-CCTCCAATTTCTGGGAAGCAG-3’ and 5’-GTTGGGACTTTGGTATTAGAA-3’.

Antibodies anti-SIN1 (D7G1A) (12860, dilution: 1:1000), anti-AKT (Pan) (40D4) (2920, dilution: 1:1000), anti-phospho-AKT (S473) (D9E) (4060, dilution: 1:1000), anti-phospho-CDK substrate motif (9477, dilution: 1:1000), anti-Rictor (D16H9) (9476, dilution: 1:1000), anti-Raptor (E6O3A) (48648, dilution: 1:1000), anti-S6K (2708), anti-phospho-S6K Thr389 (9234), anti-4EBP1 (9644), anti-phospho-4EBP1 Thr37/46 (2855) were purchased from Cell Signaling Technology (CST). Anti-USP33 (1D7) (sc-100632, dilution: 1:1000) and anti-Ub (sc-8017, dilution: 1:500) antibodies were purchased from Santa Cruz Biotechnology. Anti-Flag (F1804, dilution: 1:3000), anti-HA (H3663, dilution: 1:1000) and anti-β-actin (A1978, dilution: 1:5000) antibodies were purchased from Sigma-Aldrich. Anti-CDK1 (19532-1-AP, dilution: 1:1000) antibodies were purchased from Proteintech Group. For co-IP experiments, heavy or light chain-specific IPKine™ HRP (A25222, A25022, A25012, A25112) were from Abbkine Scientific Co.

### Western blot analysis

Cells were lysed in NETN buffer (pH 8.0, 300 mM NaCl, 20 mM Tris–HCl, 0.5%NP-40, 1 mM ethylenediaminetetraacetic acid (EDTA)) containing protease inhibitors (1× protease inhibitor cocktail (Roche), 1 mM sodium orthovanadate, 10 mM β-glycerophosphate, 1 mM phenylmethylsulfonyl fluoride, and 10 mM sodium fluoride). Proteins were separated by SDS–PAGE gel electrophoresis and transferred to PVDF membranes, then incubated with the indicated primary and secondary antibodies.

### Immunoprecipitation

Cells transfected with indicated plasmids were lysed in NETN buffer and incubated for 2 h with anti-Flag affinity gel or S-protein agarose for 4 h at 4 °C. PANC-1 and MIA PaCa-2 cells, as indicated, were lysed in NETN buffer and incubated overnight with primary antibodies together with protein A/G beads at 4 °C. The immunoprecipitates were washed three times and subjected to western blotting.

Denaturing immunoprecipitation for ubiquitination and denaturating Ni-NTA pulldown.

The cells were lysed in 100 ml 62.5 mM Tris–HCl (pH 6.8), 10% glycerol, 2% SDS, 1 mM iodoacetamide, and 20 mM NEM, boiled for 15 min, diluted 10 times with NETN buffer containing protease inhibitors, 20 mM NEM, and 1 mM iodoacetamide, then centrifuged to remove cell debris. Cell extracts were subjected to immunoprecipitation with the indicated antibodies and blotted with the indicated antibody. Denaturating Ni-NTA pulldown was performed as previously described [13].

### Tandem affinity purification and mass spectrometry analyses

In brief, tandem affinity purification in cells expressing Flag-S empty vector, Flag-S-SIN1, or Flag-S-USP33 was conducted by combining Flag-tag purification with anti-Flag affinity gel (first purification) followed by immunoprecipitation with the S-protein agarose (second purification). PANC-1 cells were transfected with Flag-S-tagged empty vector, Flag-S-SIN1, or Flag-S-USP33. Cells were treated with MG132 (10 µM) for 10 h and cell pellets were then collected and lysed in NETN buffer (pH 8.0, 300 mM NaCl, 20 mM Tris–HCl, 0.5% NP-40, 1 mM ethylenediaminetetraacetic acid (EDTA)) containing protease inhibitors (1×protease inhibitor cocktail (Roche), 1 mM sodium orthovanadate, 10 mM β-glycerophosphate, 1 mM phenylmetnylsulfonyl fluoride, and 10 mM sodium fluoride). 20 µL Anti-Flag Affinity Gel (Sigma-Aldrich) was added to cell lysates and rotated at 4 °C for 2 h. Anti-Flag immunoprecipitates were washed three times with cold NETN buffer and incubated with 100 µL 3×Flag peptide working solution at a concentration of 100 μg/mL (Sigma-Aldrich, F4799) at 4 °C for 2 h. The elution process was repeated three additional times, and the combination of eluates was diluted with NETN buffer. For the second purification step, 50 µL S-protein agarose (Merck Millipore) was added to the diluted eluates and incubated at 4 °C for 4 h. The immunoprecipitates by S-protein agarose were washed with NETN buffer three times. The beads were resuspended in 500 μL 6 M urea in PBS, 25 μL of 200 mM DTT in 25 mM NH_4_HCO_3_ buffer was added, and the reaction was incubated at 37 °C for 30 min. For alkylation, 25 μL of 400 mM IAA in 25 mM NH_4_HCO_3_ buffer was added, followed by incubation for 30 min at room temperature in the dark. The supernatant was then removed, and the beads were washed with 1 mL PBS once. For the digestion, 150 μL 2 M urea in PBS, 150 μL 1 mM CaCl_2_ in 50 mM NH_4_HCO_3,_ and 1 μL of trypsin (1.0 μg/μL) were added. The reaction was incubated at 37 °C overnight. After evaporation in SpeedVac, the samples were tested by LC–MS/MS, equipped with an EASY-nLC 1200 HPLC system and QE Plus mass spectrometer (Thermo Fisher Scientific). About 1 μg of each sample was injected into a trap column (75 μm × 2 cm, C18, 3 μm, 100 Å, 164535) and an AcclaimPepMapC18 RSLC 75 μm ID × 25 cm separation column in an EASY-Spray setting, and three injections were loaded for each sample continuously. Peptides were separated with a 70-min gradient (buffer A: 0.1% formic acid in deionized water; buffer B: 0.1% formic acid in 80% acetonitrile with a flow rate of 0.3 μL/min: 51 min of 4–28% B, 5 min of 28–38% B, 4 min of 38–90% B, 5 min of 90–4% B, 5 min of 4%B). Separated peptides were then directly analyzed on the QE Plus in a data-dependent manner. Peptides were ionized by using a spray voltage of 2.4 kV, and the ion transfer tube temperature was 320 °C, with automatic switching between MS and MS/MS scans using an exclusion duration 25 s. Mass spectra were acquired at a resolution of 60,000 with a target value of 3 × 10^6^ ions or a maximum integration time of 20 ms. The scan range was limited from 350 to 1500 *m*/*z*. Peptide S44 fragmentation was performed via higher-energy collision dissociation (HCD) with the energy set to 30%. High-resolution MS2 spectra were acquired with an exclusion duration of 25 s in the QE Plus with a maximum injection time of 40 ms at 17,500 resolution (isolation window 1.6 *m*/*z*), an AGC target value of 1 × 10^5^ and normalized collision energy of 30%. The fixed first *m*/*z* was 100, and the isolation window was 1.6*m*/*z* [14]. The raw data were processed by using Proteome Discoverer 2.5 and processed as per the default workflow. MS tolerance is 4.5 ppm, and MS/MS tolerance is 20 ppm. Searches were performed against the *Homo sapiens* uniport canonical 20,395 entries 20,210,516 nm fasta. Reversed database searches were used to evaluate the false discovery rate (FDR) of site, peptide, and protein identifications. Two missed cleavage sites of trypsin were allowed [15].

### Glutathione-S-transferase (GST) pull-down assay

Recombinant GST-USP33 or GST-SIN1 and His-CDK1, His-SIN1 proteins were expressed in *Escherichia coli* strain BL21. GST, GST-USP33, or GST-SIN1 protein was purified using Pierce Glutathione agarose. Fusion proteins were mixed for 4 h at 4 °C. Beads were washed four times, and proteins were detected by western blotting [15].

### In vitro ubiquitination assay

Cells transfected with Flag-SIN1 were treated with 10 mM MG-132 for 10 h. Proteins in the cell lysate were immunoprecipitated SIN1 with anti-Flag affinity gel, which was detected with anti-Flag antibody. The recombinant GST-tagged USP33 WT and C194S H673Q mutant (Mu) protein was expressed in *Escherichia coli* strain BL21 and purified using Pierce Glutathione Agarose. The proteins were then eluted with GST washing buffer (10 mM GSH and 50 mM Tris–HCl, pH = 8.0). The ubiquitinated SIN1 protein was then incubated with purified GST-USP33 WT and Mutant protein separately for 4 h at 4 °C, followed by western blotting analysis [15].

### In vitro kinase assay

Cells transfected with Flag-CDK1 and collected after 48 h. Proteins in the cell lysate were immunoprecipitated CDK1 with anti-Flag affinity gel. The recombinant GST-USP33 WT and 2A mutant protein was expressed in *Escherichia coli* strain BL21 and purified using Pierce Glutathione Agarose. The proteins were then eluted with GST washing buffer (10 mM GSH and 50 mM Tris–HCl, pH = 8.0) and purified with an ultrafiltration tube. Purified CDK1 affinity gel was finally incubated with purified GST-USP33 WT and 2A in kinase buffer (50 mM Tris–HCl, pH 7.4, 50 mM NaCl, 10 mM MgCl_2_, 10 mm β-glycerophosphate, 1 mm dithiothreitol (DTT), and 100 μM ATP). The reaction was carried out at 30 °C for 30 min and stopped by the addition of loading buffer. The samples were resolved by SDS–PAGE, transferred onto PVDF, and followed by western blotting analysis.

### Quantitative real-time PCR (qRT-PCR)

RNA extraction from cultured cells was performed using TRIzol reagent (Thermo Scientific, MA, USA), and then RNA was subsequently reverse transcribed to cDNA using a FastKing gDNA Dispelling RT SuperMix (Tiangen, Beijing, China). qRT-PCR analysis was performed using FastFire qPCR PreMix (SYBR Green) using primers against SIN1. All experiments were performed in triplicate with GAPDH as an internal control. All samples were normalized to *GAPDH* mRNA levels. Primer sequences are listed. *hSIN1* Forward: TCCACAGACTGCGATTCACAACC; Reverse: TCTTCAGCAAGGTCACAGGCACA. GAPDH Forward: GATCGAATTAAACCTTATCGTCGT; Reverse: GCAGCAGAACTTCCACTCGGT.

### Cell proliferation assay

PANC-1 or MIA PaCa-2 (4 × 10^4^) cells were seeded in six-well plates, and each group was in six wells. Cells for one of six wells were digested with 0.25% trypsin at 37 °C the next day. The cell pellets were collected by centrifugation (3000 rpm for 5 min), washed with PBS twice, re-suspended in PBS, and then counted in a microscope. Likewise, cells for the next 4 days are counted in a similar method [15].

### CCK-8 Assay

Cell Counting Kit-8 (HY-K0301) is used to measure the proliferation of PANC-1 and MIA PaCa-2 cells. A total of 2000 cells in a volume of 100 μL per well were cultured in four replicate wells in a 96-well plate in medium containing 10% FBS. The gemcitabine was treated with different concentrations after 24 h, and then CCK-8 reagent (15 μL) was added to incubate for 1 h after 72 h [15].

### Animal studies

For subcutaneous xenografting, PANC-1 cells (1 × 10^6^) were injected subcutaneously in mouse flanks (*n* = 6). Tumor volumes were measured three times weekly by using a vernier caliper to measure the short diameter and long diameter of the tumor. Tumor volumes were calculated using the following formula: width^2^ × length × 0.4 (mm^3^). When tumor volumes reached 100 mm^3^, mice were administered saline or RO-3306 (4 mg/kg) every two days. Gemcitabine (120 mg/kg) was administered three times weekly. After tumors had grown for a designated time, all mice were euthanized, and tumors were harvested. All animal experiments were performed in accordance with a protocol approved by the Institutional Animal Care and Use Committee of Jinan University (202207424-07).

For patient-derived tumor xenografts (PDXs), pancreatic tumors for the fifth generation of mice (P5) were purchased from Nanchang Royo Biotech Co., Ltd. PDAC tumors were cut into pieces of 3 × 3 × 3 mm^3^, and tumor tissues were pushed under the skin of mice by trochar. Tumor volumes were measured three times weekly by using a vernier caliper. When tumor volumes reached 30 mm^3^, mice were randomly divided into different groups. Lentiviruses were produced in HEK293T cells, filtered through a 0.45-µm filter, and concentrated using a PEG-8000 (DH230-1). The xenograft tumors were intratumorally injected with shScramble, shUSP33#1, shUSP33#2, shUSP33#1 + Flag-SIN1 or shUSP33#1 + Flag-USP33 WT, shUSP33#1 + Flag-USP33 2A lentivirus at a dose of 1 × 10^8^ pfu/100 μL per mouse every 3 days for three times to knockdown or overexpress USP33 expression in the tumor [16–18]. Gemcitabine (120 mg/kg) was administered three times weekly. After tumors had grown for the designated time, all mice were euthanized, and tumors were harvested. All animal experiments were performed in accordance with a protocol approved by the Institutional Animal Care and Use Committee of Jinan University (20230205-08).

### Immunohistochemical staining

PDAC pathological tissue sections were obtained from the tissue bank at The First Affiliated Hospital of Jinan University in accordance with the approval document of the Institutional Medical Ethics Committee (Ethics Approval License: JNUKY-2022-097). Tissue samples with anti-SIN1 (1:500; Proteintech Group; 15463-1-AP), anti-CDK1 (1:1000; Proteintech Group; 19532-1-AP), and anti-USP33 (1D7) (1:200; Santa Cruz Biotechnology; sc-100632) antibodies were used for immunohistochemical staining of formalin-fixed paraffin-embedded pancreatic cancer tissues were incubated at 4 °C for 12 h. The immunostaining was randomly scored by two pathologists. The IHC score was calculated by combining the quantity score (percentage of positive stained tissues) with the staining intensity score. The quantity score ranges from 0% to 24% of tissues are stained; 25–49% are positive; 50–74% are positive; and ≥75% of tissues are positive. The staining intensity with scored 1–2, categorized as low, and 3–4 as high. The score for each tissue was calculated by multiplying the quantity by the intensity score (the range of this calculation was therefore 0–4). The *χ*^2^-test and the Pearson’s correlation coefficient were used for statistical analysis of the correlation between CDK1, USP33, and SIN1.

### Kaplan–Meier survival analysis

Kaplan–Meier survival analysis was performed using the Kaplan–Meier Plotter online tool (http://kmplot.com) to evaluate the prognostic significance of SIN1 in PDAC. The survival curves and corresponding hazard ratios were calculated based on default settings. The analysis was conducted as previously described [19].

### Statistical analysis

All in vitro experiments were conducted independently three times to ensure repeatability. In the animal studies, data are expressed as the mean ± SD from six mice. GraphPad Prism software version 9.3 was used for statistical analyses. One-way ANOVA analysis followed by Tukey’s test or *t-*test was utilized to compare results with significance levels defined as ^n,s^*P* < 0.05; *P < 0.05; **P < 0.01; ***P < 0.001. The relationship of clinical data was assessed using the Chi-square test (*χ*^2^ test).

## Results

### USP33 is a key deubiquitinase that stabilizes SIN1

**Fig. 1:**
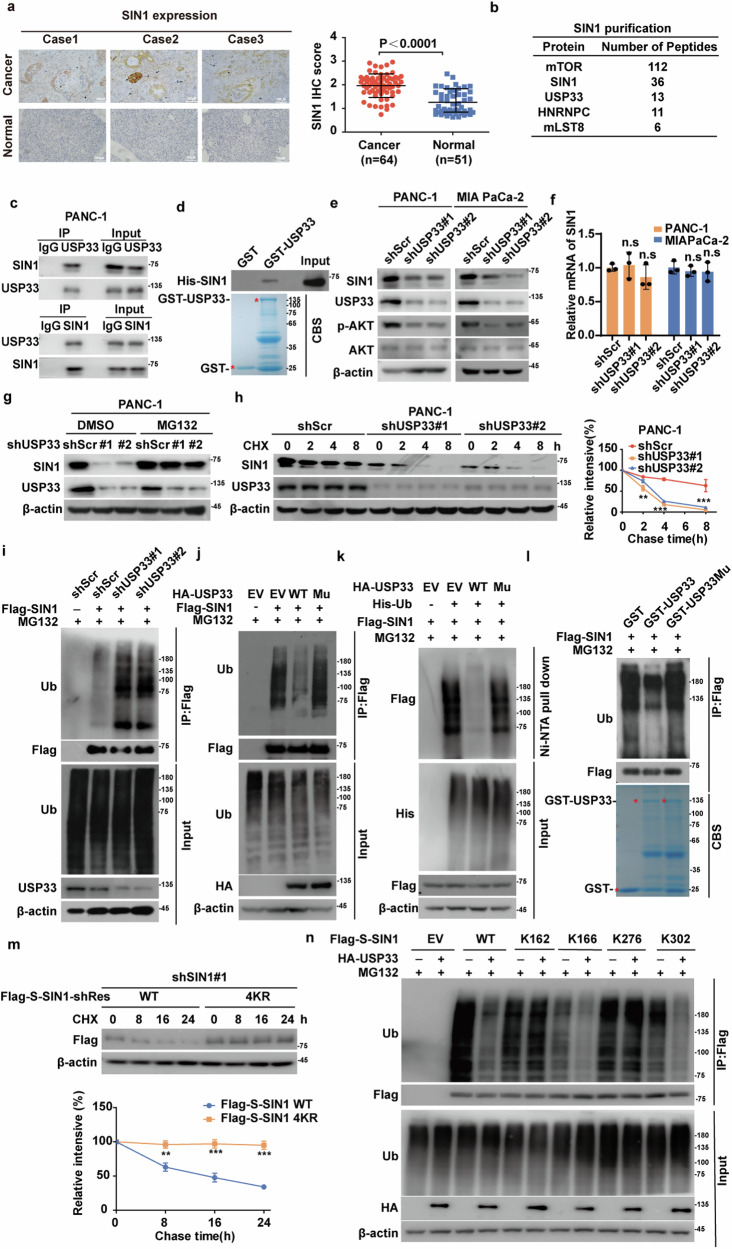
Identification of USP33 as the Bona Fide Deubiquitinase of SIN1. **a** Representative images of immunohistochemical staining of SIN1 in tumor (*n* = 64) or normal (*n* = 51) samples of PDAC (left panel). Scale bar, 100 μm. The statistical plot shows the relative expression of SIN1 in immunohistochemical staining (right panel). IHC scores were calculated as staining intensity multiplied by percent positive area. **b** List of SIN1-associated proteins identified by mass spectrometric analysis. PANC-1 cells stably expressing Flag-S-SIN1 were generated, and SIN1 immunoprecipitates were subjected to mass spectrometric analysis. **c** Cell lysates of PANC-1 were subjected to immunoprecipitation with IgG, anti-SIN1, or anti-USP33 antibodies, respectively. Western blot was performed with the indicated antibodies. **d** Purified recombinant His-SIN1 and GST or GST-USP33 were incubated in vitro as indicated. The interaction between USP33 and SIN1 was examined. CBS, Coomassie blue staining. **e** PANC-1 and MIA PaCa-2 cells stably expressing shScramble (shScr), shUAP33#1 and shUSP33#2 were generated, and western blotting was performed with the indicated antibodies. **f** *SIN1* mRNA levels in cells **e** were determined by qRT-PCR. Data shown as mean ± SD (*n* = 3). **g** PANC-1 cells stably expressing shScramble (shScr), shUAP33#1 and shUSP33#2 were treated with DMSO or MG132 (10 μM) for 10 h. Western blotting was performed. **h** Cycloheximide pulse-chase assay was performed in cells (**e**); SIN1 protein levels relative to β-actin were measured by image J. Data shown as mean ± SD (*n* = 3). **i** PANC-1 cells stably expressing shScramble (shScr), shUAP33#1 and shUSP33#2 were transfected as indicated and treated with MG132 (10 μM) for 10 h. Cell lysates were immunoprecipitated with anti-Flag affinity gel and immunoblotted as indicated. **j** PANC-1 cells were cotransfected with empty vector (EV), Flag-SIN1, HA-USP33 WT or C194S H673Q (Mu) as indicated, then were treated with MG132 for 10 h. Cell lysates were immunoprecipitated with anti-Flag affinity gel and immunoblotted as indicated. **k** Cells were cotransfected with indicated plasmids, and Ni-NTA agarose was used to pull down His-tagged ubiquitin, and the polyubiquitylated SIN1 protein was examined by western blotting. **l** PANC-1 cells were transfected with Flag-SIN1 and treated with MG132 (10 μM) for 10 h. Cell lysates were immunoprecipitated with anti-Flag affinity gel and incubated with purified GST, GST-USP33 WT, or GST-USP33 C194S H673Q (Mu) in a cell-free condition. The polyubiquitylated SIN1 protein was detected by anti-ubiquitin antibody. **m** PANC-1 cells stably expressing shSIN1#1 were transfected with Flag-S-SIN1 WT or the 4KR (K162R/K166R/K276R/K302R) mutant, and a cycloheximide pulse-chase assay was performed. The relative level of SIN1 to β-actin was measured by Image J. Data shown as mean ± SD (*n* = 3). **n** Cells expressing empty vector, Flag-S-SIN1 WT or K162, K166, K276, K302 mutants were cotransfected with empty vector or HA-USP33 and treated with MG132 (10 μM) for 10 h. Cell lysates were immunoprecipitated with anti-Flag affinity gel and immunoblotted as indicated. Data were analyzed by two-sided Student’s *t* test in (**a**, **m**), by two-sided one-way ANOVA in (**f**, **h**). Statistical significance was determined using the following p-values: *P < 0.05; **P < 0.01; ***P < 0.001.

SIN1 is a critical component of the mTORC2 complex, and its depletion impairs the assembly and function of mTORC2. Although elevated SIN1 has been identified in several cancers, its role in PDAC remains unexplored. To investigate this, we analyzed data from the public gene expression profiling interactive analysis (GEPIA) cancer database and found the *SIN1* expression is significantly higher in PDAC compared to normal tissues (Supplementary Fig. 1a). We validated these findings by IHC analysis of 64 human PDAC specimens, showing that significantly elevated SIN1 expression in tumor tissues compared to paired adjacent normal tissues (Fig. 1a). Furthermore, Kaplan–Meier plot analysis indicated that lower *SIN1* expression was correlated with better overall survival (OS) and relapse-free survival (RFS) (Supplementary Fig. 1b). We next validated therapeutic benefits of targeting SIN1 in PDAC, as knockdown of SIN1 significantly decreased pS473-AKT (a marker of mTORC2 activity) [20], inhibited cell proliferation, and increased gemcitabine sensitivity in PDAC cells (Supplementary Fig. 1c–e). These results underscore the critical role of SIN1 in PDAC progression.

To explore the mechanisms regulating SIN1 stability in PDAC, we performed tandem affinity purification and mass spectrometry analysis using PANC-1 cells stably expressing Flag-S-SIN1. Along with several known SIN1 interactors, including mTOR and mLST8 [21], we identified the deubiquitinase USP33 as a potential binding partner of SIN1 (Fig. 1b). Co-immunoprecipitation (Co‐IP) assay confirmed the endogenous interaction between USP33 and SIN1 in PANC-1 cells (Fig. 1c). Moreover, purified GST-USP33, but not GST alone, interacted with purified SIN1 under cell-free conditions (Fig. 1d). To map the binding regions, we generated a series of USP33 and SIN1 deletion mutants and conducted Co‐IP assay. As shown in Supplementary Fig. 1f, only the full‐length (FL) and the M4 (717–942aa) and M5 (811–942aa) mutants of USP33, but not other mutants, interacted with SIN1. Similarly, the full-length (FL) and M2 (138–266aa) mutants of SIN1 were capable of binding USP33 (Supplementary Fig. 1g). These data indicate that the M5 region of USP33 and the M2 region of SIN1 are critical for their interaction.

Given that no direct deubiquitinase of SIN1 has been identified so far, we next investigated whether USP33 plays a role in regulating SIN1 stability and its oncogenic function. Depletion of USP33 in PANC-1 and MIA PaCa-2 cells significantly reduced SIN1 protein levels and inhibited pS473-AKT phosphorylation (Fig.1e). As shown in Supplementary Fig. 1h, the depletion of USP33 also led to a significant reduction of overexpressed Flag-SIN1 levels. Importantly, USP33 depletion did not affect *SIN1* mRNA level, while proteasome inhibition with MG132 rescued the decreased SIN1 protein levels in USP33-depleted cells (Fig. 1f, g and Supplementary Fig. 1i). Cycloheximide pulse-chase assay revealed that SIN1 was less stable in USP33-depleted cells (Fig. 1h and Supplementary Fig. 1j). Furthermore, depletion of USP33 markedly increased SIN1 ubiquitination levels (Fig. 1i), while USP33 WT, but not the catalytically inactive USP33 mutant (C194S-H673Q, Mu), reduced SIN1 ubiquitination (Fig. 1j, k). In vitro assay confirmed that purified GST-USP33 WT, rather than the inactive mutant, efficiently cleaved polyubiquitinated SIN1 (Fig. 1l). These results indicate that USP33 specifically deubiquitinates and stabilizes SIN1 in PDAC.

To identify the specific ubiquitin linkage of SIN1 targeted by USP33, we found that SIN1 was polyubiquitinated through both K48- and K63-specific chains, but USP33 preferentially cleaved the K48-linked polyubiquitin chain (Supplementary Fig. 1k, l). We then introduced point mutation at potential ubiquitination sites in SIN1 based on the PhosphoSitePlus database. As shown in Fig. 1m and Supplementary Fig. 1m, mutants K162R, K166R, K276R, and K302R exhibited reduced SIN1 ubiquitination, while the 4KR mutant (K162R/K166R/K276R/K302R) significantly increased SIN1 stability. We next generated several SIN1 mutants containing a single intact lysine residue, replacing all other lysines with arginines. As shown in Fig. 1n, USP33 WT effectively decreased the ubiquitination of the SIN1 mutants K166 and K302, but had no effect on K162 and K276 mutants. These findings suggest that USP33 selectively deubiquitinates the K48-specific polyubiquitin chains at lysine residues K166 and K302, thereby stabilizing SIN1.

### USP33 promotes PDAC progression through stabilizing SIN1

**Fig. 2:**
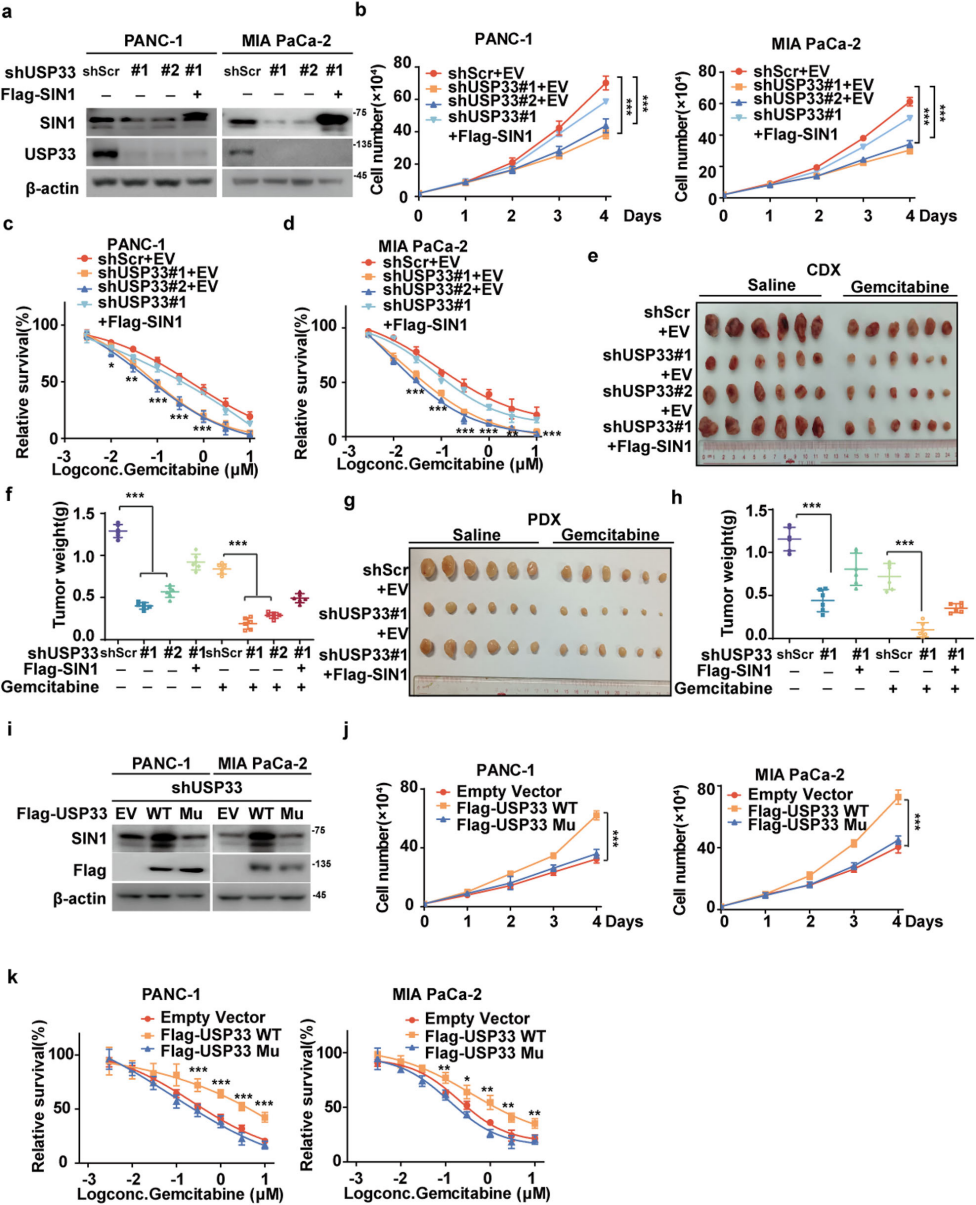
USP33 promotes PDAC progression through stabilizing SIN1. **a** PANC-1 and MIA PaCa-2 cells stably expressing shScramble (shScr), shUSP33#1 and shUSP33#2 were transfected with empty vector (EV) or Flag-SIN1, and western blotting was performed with the indicated antibodies. **b** Cell proliferation of PANC-1 and MIA PaCa-2 cells, as in (**a**) was examined. Data shown as mean ± SD (*n* = 3). **c**, **d** PANC-1 and MIA PaCa-2 cells as in (**a**) were treated with indicated concentrations of gemcitabine, and cell survival was determined. Data shown as mean ± SD (*n* = 4). **e**, **f** PANC-1 cells as in (a) were subcutaneously implanted into nude mice (5–6 weeks, *n* = 6). When tumors reached around 150–200 mm^3^ in size, mice were treated with saline or gemcitabine (120 mg/kg). Tumors were collected (**e**), and weights were measured (**f**). Data shown as mean ± SD (*n* = 6). **g**, **h** PDAC patient-derived tumor xenograft (PDX) were subcutaneously implanted into nude mice (5–6 weeks, *n* = 6). When tumors reached around 150–200 mm^3^ in size, mice were treated with saline or gemcitabine (120 mg/kg). Tumors were collected (**g**) and weights were measured (**h**). Data shown as mean ± SD (*n* = 6). **i** PANC-1 and MIA PaCa-2 cells stably expressing shUSP33 were transfected with empty vector (EV), Flag-USP33 WT or USP33 C194S H673Q (Mu), and western blotting was performed with the indicated antibodies. **j** Cell proliferation of PANC-1 and MIA PaCa-2 cells, as in (**i**) was examined. Data shown as mean ± SD (*n* = 3). **k** PANC-1 and MIA PaCa-2 cells as in (**i**) were treated with indicated concentrations of gemcitabine, and cell survival was determined. Data shown as mean ± SD (*n* = 4). Data were analyzed by two-sided one-way ANOVA in (**b–d**, **f**, **h**, **j**, **k**). Statistical significance was determined using the following p-values: *P < 0.05*;* **P < 0.01*;* ***P < 0.001.

Previous studies have shown that activation of the mTORC2-AKT pathway can drive tumor growth and reduce cellular response to chemotherapies [22]. We next investigated whether USP33 promotes tumor progression in PDAC in a SIN1-dependent manner. As shown in Fig. 2a–d, depletion of USP33 in PANC-1 and MIA PaCa-2 cells significantly reduced SIN1 protein levels, suppressed cellular proliferation, and increased sensitivity to gemcitabine, whereas reconstitution of SIN1 in USP33-deficient cells markedly rescued these effects. These findings were further validated in vivo using both PANC-1 cell-based and patient-derived xenograft (PDX) models (Fig. 2e–h). Additionally, reconstitution of USP33 WT in endogenous USP33-deficient PDAC cells significantly increased SIN1 protein levels and promoted SIN1-dependent malignant phenotypes (Fig. 2i–k). In contrast, the catalytically inactive USP33 mutant did not affect SIN1 protein levels or its oncogenic functions. Taken together, these results demonstrate that USP33 promotes PDAC progression primarily through deubiquitinating and stabilizing SIN1, positioning USP33 as a promising therapeutic target in PDAC.

### CDK1 binds and phosphorylates USP33 at Ser650 and Ser843

**Fig. 3:**
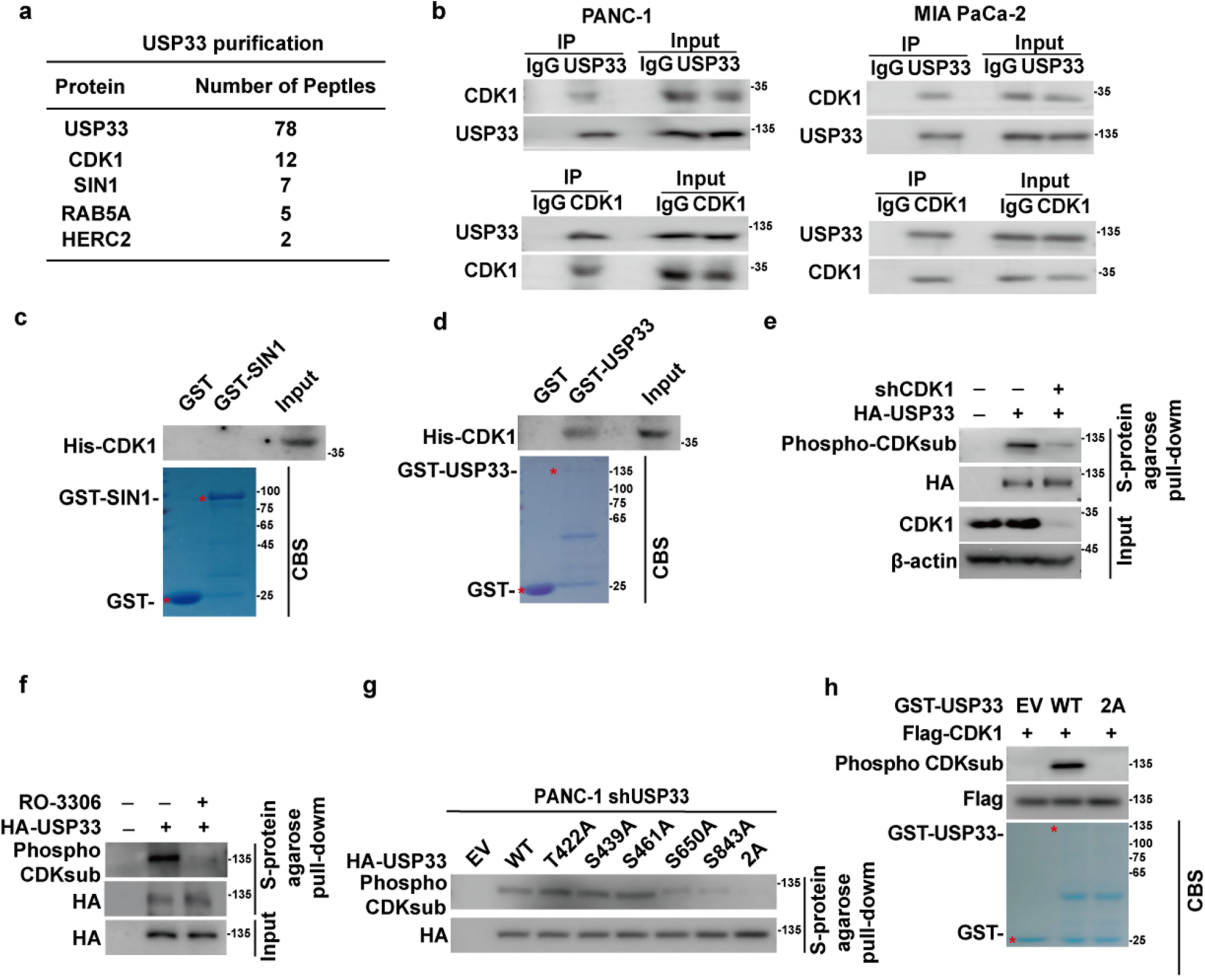
CDK1 phosphorylates USP33 at Ser650, Ser843. **a** List of USP33-associated proteins identified by mass spectrometric analysis. PANC-1 cells stably expressing Flag-S-USP33 were generated, and USP33 immunoprecipitates were subjected to mass spectrometric analysis. **b** PANC-1 and MIA PaCa-2 cell lysates were subjected to immunoprecipitation with IgG, anti-USP33, or anti-CDK1 antibodies. The immunoprecipitates were blotted with the indicated antibodies. **c** Purified recombinant His-CDK1 and GST or GST-SIN1 were incubated in vitro as indicated. The interaction between CDK1 and SIN1 was examined. CBS, Coomassie blue staining. **d** Purified recombinant His-CDK1 and GST or GST-USP33 were incubated in vitro as indicated. The interaction between CDK1 and USP33 was examined. CBS Coomassie blue staining. **e** Cells were transfected with empty vector, HA-S-USP33, and treated with DMSO or RO-3306 (5 μM). Cell lysates were pulled down by S-protein agarose and the phosphorylation of USP33 was examined by phospho-CDK substrate antibody. **f** Cells were transfected with empty vector, HA-S-USP33, and treated with DMSO or RO-3306 (5 μM). Cell lysates were pulled down by S-protein agarose. The immunoprecipitates were blotted with the indicated antibodies. **g** Endogenous USP33-deficient PANC-1 cells were transfected with empty vector, HA-S-USP33, the single mutants, the 2A mutants, or indicated plasmids and then treated with MG132 (10 μM) for 10 h. Cell lysates were pulled down by S-protein agarose and the phosphorylation of USP33 was examined by using phospho-CDK substrate antibody. **h** CDK1 phosphorylates USP33 in vitro. Bacterial purified GST, GST-USP33 WT, or GST-USP33 2A mutant incubated with active CDK1. Western blotting was performed and the phosphorylation of USP33 was examined using phospho-CDK substrate antibody.

Our results suggest that USP33 promotes tumor progression in PDAC by stabilizing SIN1 through inhibiting its polyubiquitination and subsequent degradation. However, the molecular mechanisms regulating USP33 in PDAC remain poorly understood, and strategies for directly targeting USP33 have not yet been developed. To explore upstream regulatory mechanisms of USP33, we performed the tandem affinity purification and mass spectrometry analysis by using PANC-1 cells stably expressing Flag-S-USP33. As shown in Fig. 3a, b, we identified several interactors of USP33, including CDK1 and SIN1. We further confirmed the interaction between CDK1 and USP33 in PDAC cells. In a cell-free system, purified GST-USP33, but not GST or GST-SIN1 protein, bound to His-CDK1, suggesting a direct interaction between CDK1 and USP33 (Fig. 3c, d).

CDK1 has been shown to phosphorylate and regulate distinct functions of several substrates, including disruptor of telomeric silencing 1-like (DOT1L), SRY-box transcription factor 2 (SOX2), and pyruvate dehydrogenase kinase 1 (PDK1), in various cancers [23–25]. We next explored whether CDK1 may phosphorylate USP33 and affect its tumor-promoting function in PDAC cells. As shown in Fig. 3e, f, phosphorylation of USP33 was detected in PANC-1 cells using a phospho-CDK substrate antibody. Genetic ablation or pharmacological inhibition of CDK1 with RO-3306 significantly reduced this phosphorylation. To identify the specific phosphorylation sites on USP33 targeted by CDK1, we analyzed its amino acid sequence and identified several potential CDK1 consensus phosphorylation motifs [26], including Thr422, Ser439, Ser461, Ser650, and Ser843 (Supplementary Fig. 2a). Mutation of USP33 at S650A or S843A, but not at T422A, S439A, or S461A, partially decreased phosphorylation. The double mutant (S650A/S843A, 2A) almost completely abolished phosphorylation (Fig. 3g). To further confirm that CDK1 directly phosphorylates USP33, we performed an in vitro kinase assay, where GST-fused USP33 WT or the 2A mutant was incubated with active CDK1. As shown in Fig. 3h, active CDK1 phosphorylated GST-fused USP33 WT in vitro, while the 2A mutant exhibited significantly reduced phosphorylation. These results suggest that CDK1 regulates the phosphorylation of USP33 at Ser650 and Ser843.

### CDK1 regulates SIN1 protein stability, cell growth, and sensitivity to chemotherapy

**Fig. 4:**
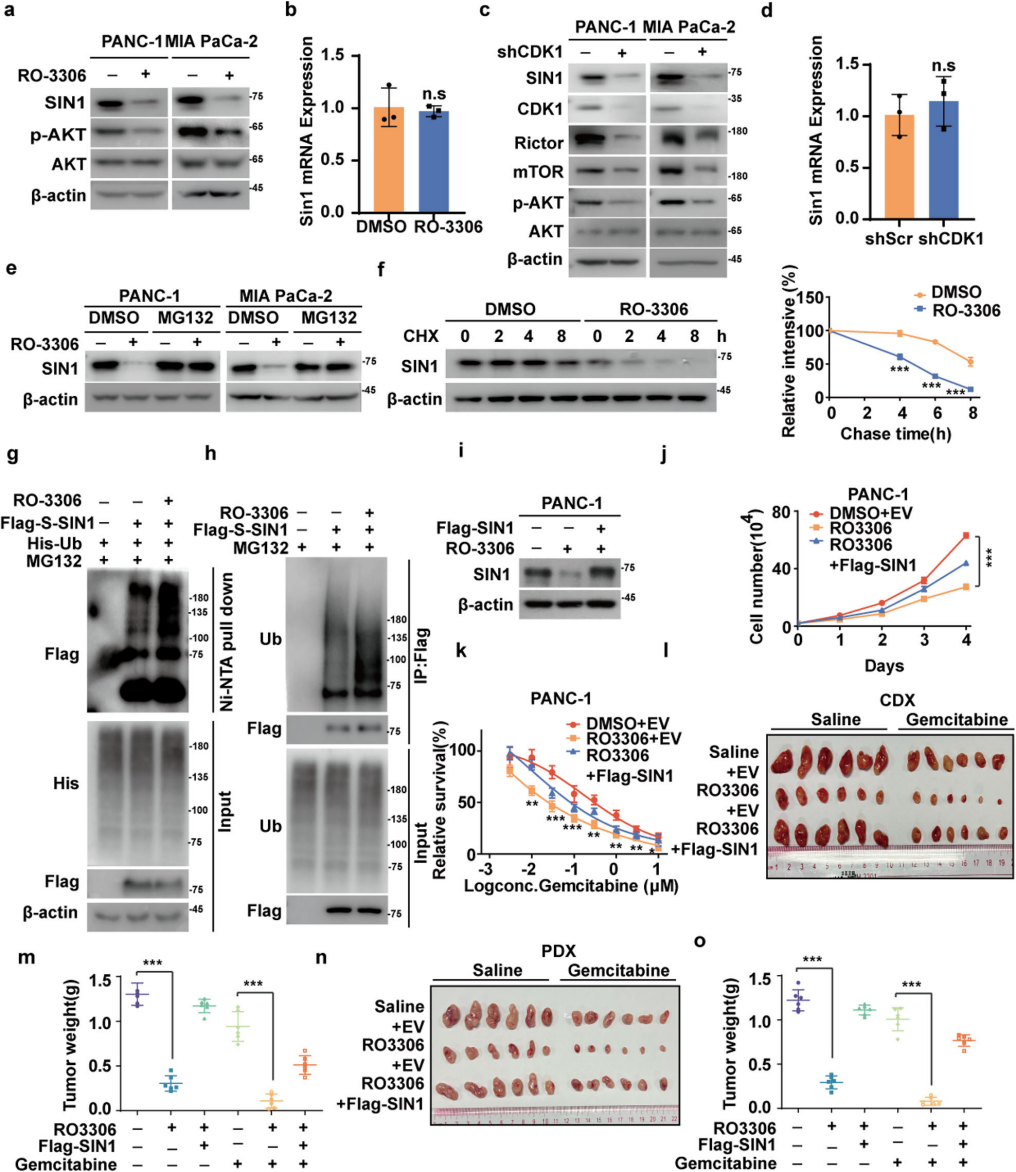
CDK1 inhibition induces SIN1 degradation and suppresses PDAC progression. **a** PANC-1 and MIA PaCa-2 cells were treated with DMSO or RO-3306(5 μM) for 24 h, and western blotting was performed. **b** Total RNA was isolated from cells in (**a**). The expression of *SIN1* mRNA in cells was determined by qRT-PCR. Data shown as mean ± SD (*n* = 3). **c** PANC-1 and MIA PaCa-2 cells stably expressing shScramble (shScr), shCDK1 were generated, and western blotting was performed with the indicated antibodies. **d** Total RNA was isolated from cells in (**c**). The expression of *SIN1* mRNA in cells was determined by qRT-PCR. Data shown as mean ± SD (*n* = 3). **e** Cells were pretreated with DMSO or RO-3306 (5 μM) for 24 h and then treated with DMSO or MG132 (10 μM) for 10 h. Western blotting was performed. **f** Cycloheximide pulse-chase assay was performed in the presence or absence of RO-3306. The relative level of SIN1 to β-actin was measured by image J. Data shown as mean ± SD (*n* = 3). **g** Cells were transfected with empty vector (EV) or indicated plasmids and treated with DMSO or RO-3306 (5 μM), followed by MG132 treatment. Ni-NTA beads were used to pulldown His-tagged ubiquitin, and polyubiquitylated SIN1 was examined. **h** Cells were transfected with empty vector (EV) or indicated plasmids and treated with DMSO or RO-3306 (5 μM), followed by MG132 treatment. Cell lysates were immunoprecipitated with anti-Flag affinity gel and western blotting as indicated. **i** PANC-1 cells pretreated with DMSO, RO-3306, or RO-3306 transfected with Flag-SIN1 were generated, and western blotting was performed with the indicated antibodies. **j** Cell proliferation assay was performed in PANC-1 cells as in (**i**). Data shown as mean ± SD (*n* = 3). **k** Cells as in (**i**) were treated with indicated concentrations of gemcitabine, and cell survival was determined. Data shown as mean ± SD (*n* = 4). **l, m** PANC-1 cells as in (**i**) were subcutaneously implanted into nude mice (5–6 weeks, *n* = 6). When tumors reached around 150–200 mm^3^ in size, mice were administered saline or RO-3306 (4 mg/kg) every two days. Gemcitabine (120 mg/kg) was administered three times weekly. Tumors were collected (**l**) and weights were measured (**m**). Data shown as mean ± SD (*n* = 6). **n**, **o** PDAC PDX were subcutaneously implanted into nude mice (5–6 weeks, *n* = 6). When tumors reached around 150–200 mm^3^ in size, mice were administered saline or RO-3306 (4 mg/kg) every 2 days. Gemcitabine (120 mg/kg) was administered three times weekly. Tumors were collected (*n*) and weights were measured (**o**). Data shown as mean ± SD (*n* = 6). Data were analyzed by two-sided Student’s *t* test in (**b**, **d**, **f**), by two-sided one-way ANOVA in (**j**, **k**, **m**, **o**). Statistical significance was determined using the following *p*-values: *P < 0.05; **P < 0.01; ***P < 0.001.

Given that CDK1 phosphorylates USP33, which in turn stabilizes SIN1, we hypothesized that CDK1 might also regulate the stability and function of SIN1 in PDAC. As shown in Fig. 4a–d and Supplementary Fig. 3a, b, depletion or inhibition of CDK1 in PANC-1 and MIA PaCa-2 cells led to a significant reduction in SIN1 protein levels, without affecting its mRNA expression. In addition, pharmacological inhibition of CDK1 using RO-3306 also led to a significant decrease in Flag-SIN1 levels (Supplementary Fig. 3c). In contrast, treatment with the proteasome inhibitor MG132 rescued the decrease in SIN1 protein levels caused by CDK1 depletion or inhibition, indicating that CDK1 regulates SIN1 stability through a proteasome-dependent mechanism (Fig. 4e and Supplementary Fig. 3d). Additionally, a cycloheximide pulse-chase assay revealed that SIN1 protein stability was compromised in cells treated with RO-3306 or in CDK1-depleted cells (Fig. 4f and Supplementary Fig. 3e). This decreased stability correlated with an increase in SIN1 ubiquitination (Fig. 4g, h and Supplementary Fig. 3f), further supporting the notion that CDK1 stabilizes SIN1 via the proteasome pathway.

Based on these findings, we next explored the impact of CDK1 inhibition on SIN1-dependent malignant phenotypes in PDAC. As shown in Fig. 4i–k and Supplementary Fig. 3g–l, depletion or inhibition of CDK1 in PANC-1 and MIA PaCa-2 cells markedly reduced SIN1 protein levels, inhibited cell proliferation, and enhanced cellular sensitivity to gemcitabine. Importantly, reconstitution of SIN1 largely rescued such phenotypic alterations, indicating that CDK1’s effects on cell proliferation and chemotherapy sensitivity are primarily mediated through its regulation of SIN1 (Fig. 4i–k; Supplementary Fig. 3g–l). To further validate these observations, similar results were obtained in both PDAC cell line xenograft and PDX models (Fig. 4l–o). These data demonstrate that the tumor-suppressive effects of CDK1 inhibition in PDACs are primarily driven by destabilization of SIN1, leading to impaired downstream effectors such as AKT.

### CDK1-mediated phosphorylation of USP33 is pivotal for SIN1 stability and PDAC progression

**Fig. 5:**
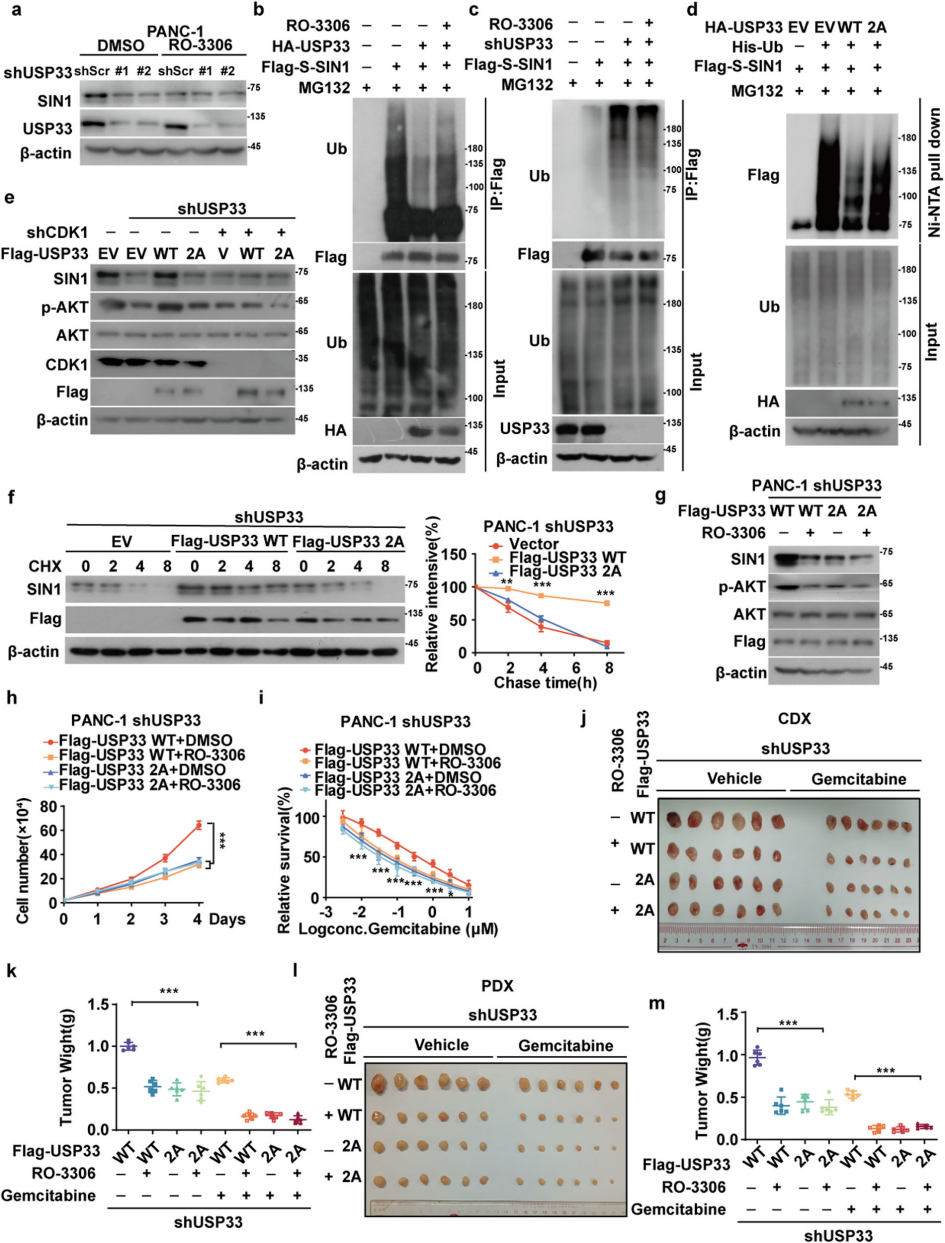
CDK1-mediated phosphorylation of USP33 is pivotal for SIN1 stability and PDAC progression. **a** SIN1 and USP33 levels in PANC-1 cells expressing shScramble (shScr) or shUSP33 treated with DMSO or RO-3305 (5 μM) were examined. **b** Cells were transfected as indicated and treated with DMSO or RO-3305 (5 μM), followed by MG132 treatment. Polyubiquitylated SIN1 was examined. **c** Cells stably expressing shScramble (shScr), shUSP33#1, or shUSP33#2 were transfected with the indicated plasmids and treated with DMSO or RO-3306. Polyubiquitylated SIN1 was examined. **d** Cells were cotransfected with empty vector, Flag-SIN1, and indicated plasmids. His-tagged ubiquitin was pulled down by Ni-NTA beads, and polyubiquitylated SIN1 was examined. **e** PANC-1 cells stably expressing shScr or shCDK1 were transfected with empty vector, Flag-USP33 WT, or the 2A mutant, and western blotting was performed. **f** PANC-1 cells expressing shUSP33 were transfected with empty vector, Flag-USP33 WT, or the 2A mutant, and a cycloheximide pulse-chase assay was performed. Data shown as mean ± SD (*n* = 3). **g** PANC-1 cells with endogenous USP33 deficiency were transfected with the indicated plasmids, and western blotting was performed. **h** Cell proliferation assay was performed in PANC-1 cells. Data shown as mean ± SD (*n* = 3). **i** Cells as in (**g**) were treated with indicated concentrations of gemcitabine, and cell survival was determined. Data shown as mean ± SD (*n* = 4). **j**, **k** PANC-1 cells as in (**g**) were subcutaneously implanted into nude mice (5–6 weeks, *n* = 6). When tumors reached around 150–200 mm^3^ in size, mice were treated with saline or gemcitabine (120 mg/kg) was administered three times weekly. Tumors were collected (**j**) and weights were measured (**k**). Data shown as mean ± SD (*n* = 6). **l**, **m** PDAC PDX were subcutaneously implanted into nude mice (5–6 weeks, *n* = 6). When tumors reached around 150–200 mm^3^ in size, mice were treated with saline or gemcitabine (120 mg/kg) was administered three times weekly. Tumors were collected (**l**) and weights were measured (**m**). Data shown as mean ± SD (*n* = 6). Data were analyzed by two-sided one-way ANOVA in (**f**, **h**, **i**, **k**, **m**). Statistical significance was determined using the following *p*-values: *P < 0.05*;* **P < 0.01*;* ***P < 0.001.

To further explore the role of CKD1 in regulating SIN1, we investigated whether USP33 mediates this effect in PDAC. As shown in Fig. 5a, depletion of USP33 or inhibition of CDK1 significantly reduced SIN1 protein levels. Notably, the combined depletion of USP33 and inhibition of CDK1 did not result in any further reduction in SIN1 protein levels, suggesting that USP33 and CDK1 may act within the same pathway. Additionally, overexpression of USP33 dramatically decreased the ubiquitination of SIN1, an effect that was significantly blocked by treatment with RO-3306 (Fig. 5b). In contrast, knockdown of USP33 markedly increased SIN1 ubiquitination, which was not substantially altered by CDK1 inhibition (Fig. 5c). These findings indicate that CDK1 activation stabilizes SIN1 through USP33, and CDK1 plays a crucial role in the deubiquitination process of SIN1 by USP33 in PDAC.

We next examined how CDK1-mediated phosphorylation of USP33 affects its ability to stabilize SIN1. As shown in Supplementary Fig. 4a, genetic ablation or pharmacological inhibition of CDK1 with RO-3306 significantly reduced the binding between USP33 and SIN1 in PANC-1 cells. Additionally, overexpression of the USP33 2A mutant resulted in much weaker binding to SIN1 compared to USP33 WT (Supplementary Fig. 4b). We then assessed the impact of CDK1-mediated phosphorylation on the ubiquitination of SIN1 mutants. As shown in Fig. 5d, overexpression of USP33 WT significantly decreased the SIN1 ubiquitination level, whereas the 2A mutant failed to do so. Furthermore, in USP33-deficient PNAC-1 cells, the reduced SIN1 protein levels could be largely rescued by the reconstitution of USP33 WT, but not the 2A mutant (Fig. 5e). Moreover, reconstitution of USP33 WT in these cells dramatically increased the half-life of SIN1 compared to the 2A mutant (Fig. 5f). These results suggest that CDK1-mediated phosphorylation of USP33 is essential for its activity to regulating the ubiquitination and degradation of SIN1.

To assess the functional consequence of CDK1-mediated phosphorylation of USP33, we examined its role in SIN1-dependent malignant processes. Reconstitution of USP33 WT in endogenous USP33-deficient PANC-1 and MIA PaCa-2 cells significantly increased cell proliferation and decreased sensitivity to gemcitabine, compared to cells expressing the USP33 2A mutant (Fig. 5g–i and Supplementary Fig. 4c–e). In vivo, similar results were observed in PANC-1-derived xenograft and PDX models (Fig. 5j–m). These results demonstrate that CDK1-mediated phosphorylation of USP33 is crucial for stabilizing SIN1 and promoting SIN1-dependent tumor progression in PDAC.

### Correlations between CDK1, USP33, and SIN1 expression in PDAC samples

**Fig. 6:**
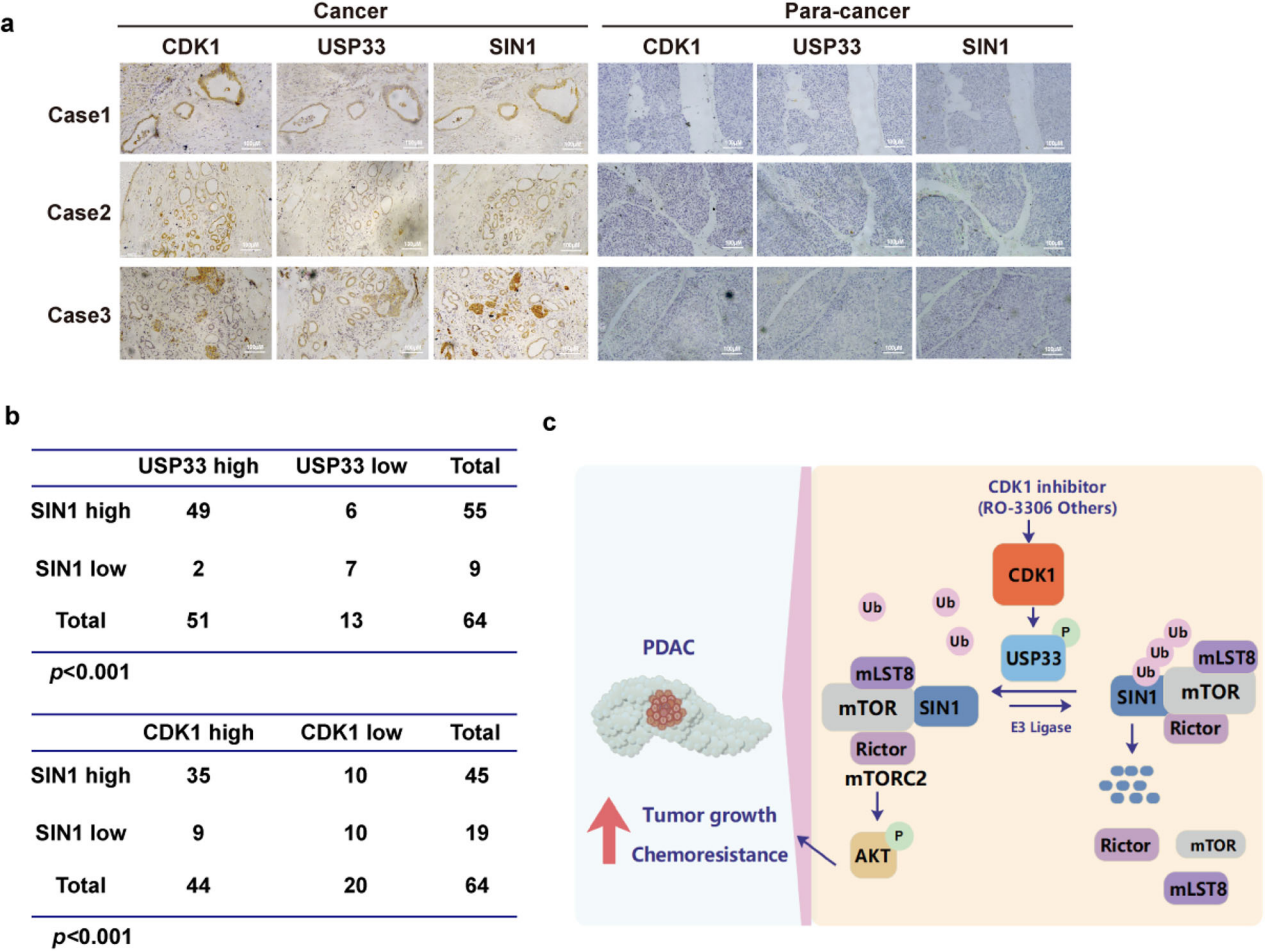
The expression of SIN1 positively correlates with USP33 and CDK1 in PDAC. **a** Representative images of immunohistochemical staining of USP33, CDK1, and SIN1 in tumor samples of PDAC (*n* = 64). **b** Positive correlation of SIN1 expression with USP33 or CDK1 was shown. Statistical analyses were performed with the Fisher exact test. **c** The working model to illustrate that CDK1 phosphorylation-dependent activation of USP33 regulates tumor growth and chemoresistance through SIN1.

We next explored the clinical relevance of the CDK1-USP33-SIN1 axis by analyzing protein expressions of CDK1, USP33, and SIN1 in 64 PDAC specimens. As shown in Fig. 6a, the expressions of CDK1, USP33, and SIN1 were significantly upregulated in PDAC tissues compared to adjacent non-cancerous tissues. Notably, we observed a positive correlation between USP33 and SIN1 expression in PDAC samples. Specifically, ~76.56% of samples with high USP33 expression also exhibited high SIN1 expression. Additionally, SIN1 expression was positively correlated with CDK1 expression, with 54.68% of samples showing high SIN1 levels also having high CDK1 expression (Fig. 6b). These findings underscore the clinical significance of the CDK1-USP33-SIN1 axis, suggesting that it may serve as a potential therapeutic target for regulating tumor growth and chemosensitivity in PDAC (Fig. 6c).

## Discussion

One critical feature of many solid tumors, including PDAC, is the overactivation of AKT, a key signaling pathway that drives tumor growth and chemoresistance. In PDAC, more than half tumors exhibit aberrant AKT activation, which strongly correlates with poor survival outcomes [27]. However, translating AKT inhibition into clinical practice has proven challenging due to issues such as limited selectivity, significant toxicities, tumor heterogeneity, and the absence of reliable response biomarkers. For instance, a phase III trial combining Ipatasertib with paclitaxel showed no improvement in efficacy for PIK3CA/AKT1/PTEN-altered HR^+^-HER2^−^ unresectable locally advanced or metastatic breast cancer [28]. Conversely, the ATP-competitive AKT inhibitor capivasertib, when combined with fulvestrant, significantly improved progression-free survival in HR^+^-HER2^−^ advanced breast cancer patients, particularly those who experienced disease progression during or after previous endocrine therapy, with or without a CDK4/6 inhibitor [29]. Despite these findings, the full potential of capivasertib and other AKT inhibitors in treating other cancers remains to be validated in further preclinical and clinical trials. This highlights the need to identify upstream regulatory mechanisms that sustain AKT activation, providing alternative therapeutic strategies.

Accumulating evidence highlights the essential role of SIN1 in the regulation of mTORC2, the only known kinase responsible for phosphorylation of AKT at Ser473 [30, 31]. SIN1 acts as a key scaffolding protein, forming a complex with Rictor and mTOR to facilitate the complex assembly. Additionally, SIN1 determines the substrate specificity of the mTOR catalytic subunit, enabling proper substrate recognition and activation [21]. Moreover, the Pleckstrin Homology (PH) domain of SIN1 binds to Ptdlns (3–5) P_3_, relieving inhibition of the mTOR kinase and promoting mTORC2 translocation to the membrane [31]. This translocation is crucial for the phosphorylation of its physiological substrates, such as AKT, which is also recruited to the membranes via its own PH domain. Given the frequent dysregulation of mTORC2 in human cancers and the lack of specific mTORC2 inhibitors, SIN1 presents a promising target for mTORC2 inhibition [32]. However, directly targeting SIN1 within mTORC2 remains challenging due to its lack of enzymatic activity and the absence of compounds that disrupt SIN1 interaction with other mTORC2 components in cancer [33]. Since SIN1 is aberrantly elevated in various cancers [12, 34], understanding the mechanisms that regulate its stability is crucial for developing potential therapeutic strategies. In this study, we reveal several unexpected findings with important implications. Specifically, we demonstrate a previously uncharacterized tumor-promoting activity of the CDK1–USP33 axis in PDAC through stabilizing SIN1, a key mediator of mTORC2-AKT activation. We show that CDK1-catalyzed phosphorylation of USP33 is a critical post-translational modification controlling SIN1 stability and its oncogenic activity. Moreover, our preclinical data demonstrate that targeting the CDK1-USP33 axis effectively suppresses tumor progression in PDAC via destabilizing SIN1 (Fig. 6c).

USP33 has been shown to exhibit either oncogenic or tumor-suppressive roles, depending on the tumor context. For instance, USP33 deubiquitinates and stabilizes HIF-2α, promoting the hypoxia response in glioma stem cells [35], and its elevated expression accelerates ovarian cancer progression by stabilizing CBX2 [36]. On the other hand, in a hepatocyte-specific knockout mouse model, USP33-deficient mice showed increased sensitivity to DEN-induced hepatocarcinogenesis, suggesting that USP33 may possess anti-tumor activity through regulating p53 stability and activity [37]. Additionally, USP33 is required for Slit signaling to inhibit breast cancer cell migration [38]. However, the precise mechanism governing USP33 function and regulation in PDAC is not fully understood. Our study provides the first evidence that USP33 acts as the deubiquitinase of SIN1, activating the mTORC2–AKT pathway and promoting PDAC progression. Several key findings support this conclusion. First, our mass spectrometry analysis identified USP33 as a bona fide deubiquitinase of SIN1, showing that USP33 directly interacts with SIN1 and reduces its ubiquitination, thereby stabilizing SIN1 (Fig. 1 and Supplementary Fig. 1). Second, genetic ablation of USP33 significantly attenuates mTORC2-AKT activation and enhances cellular sensitivity to gemcitabine in both in vitro and in vivo models (Fig. 2). We further demonstrate that the tumor-promoting activity of USP33 in PDAC is largely dependent on its ability to stabilize SIN1 (Fig. 2). Clinically, we observe a positive correlation between USP33 and SIN1 expression in PDAC samples, suggesting that USP33 may serve as a therapeutic target to manage chemoresistance in PDAC by promoting SIN1 stability and functions (Fig. 6). However, it is important to note that we cannot rule out the involvement of additional mechanisms in USP33-regulated tumor progression, as the ectopic expression of SIN1 did not fully rescue functional alterations caused by USP33 depletion in PDAC cells (Fig. 2e–h). Previous reports have shown that USP33 promotes metastasis in hepatocellular carcinoma by deubiquitinating transcription factor (SP1) and upregulating cell-surface receptor c-mesenchymal–epithelial transition factor (c-MET) expression [39]. In prostate cancer, USP33 overexpression prevents the degradation of the phosphatase dual-specificity phosphatase 1 (DUSP1), impairing c-jun N-terminal kinase (JNK) activation and apoptosis, which contributes to docetaxel resistance [40]. USP33 is also reported to enhance the protein stability of transforming growth factor beta receptor 2 (TGFBR2) by removing the K63-linked ubiquitin chains on TGFBR2, thereby enhancing transforming growth factor-beta (TGF-beta) signaling and promoting pancreatic cancer cell migration and invasion [41]. Further investigations into the involvement of other USP33 substrates, in addition to SIN1, will be necessary to better understand its role in PDAC chemoresistance.

Our study provides the rationale for targeting USP33 as a potential therapeutic strategy to overcome chemoresistance in PDAC. However, the regulation of USP33 expression or activity in human cancer is not well understood. It was reported that TGF-β signaling targeted gene zinc finger E-box-binding homeobox 1 (ZEB1) promoted the transcription of USP33 through binding to the 622–632 site of USP33 promoter [41]. Additionally, USP33 is preferentially induced in glioma stem cells by hypoxia, although no change in USP33 transcription is observed in response to hypoxia. Some E3 ligases, such as HERC2 and β-TrCP [42, 43], have been reported to regulate USP33 expression through ubiquitination and degradation. However, the mechanisms regulating USP33’s activity toward its substrates in cancer contexts remain largely unexplored.

CDK1 is known to phosphorylate not only its canonical cell cycle regulators but also non-canonical substrates, including B-Raf Proto-Oncogene, Serine/Threonine Kinase (BRAF), androgen receptor (AR), tumor protein 53 (p53) and hypoxia inducible factor 1-alpha (HIF1α), thereby playing critical roles in cell cycle progression, apoptosis, transcription, protein transport, and other cellular process [44–46]. In this study, we reveal that the deubiquitination of SIN1 by USP33 is positively regulated by the protein kinase CDK1 in PDAC. First, we demonstrate that CDK1 binds and phosphorylates USP33 at Ser650 and Ser843, and the phosphorylation-deficient 2A mutation of USP33 failed to undergo phosphorylation (Fig. 3 and Supplementary Fig. 2). Second, genetic ablation or inhibition of CDK1 in PDAC cells increased the ubiquitination and degradation of SIN1, which in turn blocked SIN1-driven phenotypes, including cell proliferation and chemoresistance (Fig. 4 and Supplementary Fig. 3). Third, CDK1-mediated phosphorylation was essential for USP33 binding to SIN1 and promoting tumor progression (Fig. 5). Importantly, histological analysis revealed a strong correlation between high expression of CDK1 and SIN1 in human PDAC specimens, which was associated with poor patient survival [41, 47], highlighting the clinical relevance of targeting the CDK1-USP33-SIN1 axis in PDAC (Fig. 6). Futhermore, emerging studies have implicated CDK1 in the pathogenesis of PDAC, where it is frequently overexpressed and associated with poor overall survival [48]. These findings suggest that CDK1 may serve as both a prognostic biomarker and a potential therapeutic target in PDAC.

Importantly, previous studies have reported that CDK1 inhibitor RO-3306 exhibits tumor-selective cytotoxicity, demonstrating up to a fourfold greater cell-killing efficacy in bladder carcinoma and laryngeal squamous cell carcinoma models compared to normal epithelial cells [49]. Furthermore, systemic administration of the multi-CDK inhibitor AT7519 (targeting CDK1/2/7/9) has shown favorable tolerability in pancreatic cancer xenograft models, with no significant adverse effects reported [50]. Despite these promising findings, several limitations must be considered. To date, no CDK1-specific inhibitors have received clinical approval. Compounds such as RO-3306 (NCT03579836) and BEY-1107 remain in early-phase clinical trials [51]. Therefore, comprehensive clinical evaluation is necessary to determine the safety, tolerability, and efficacy of CDK inhibition in cancers, including PDAC. Additionally, given CDK1’s essential role in normal cell cycle control, systemic inhibition may pose risks of off-targets or toxicity [52]. A deeper understanding of the differential role of CDK1 in cancer versus normal cells is critical for optimizing therapeutic windows and minimizing adverse effects. The development of tumor-specific delivery systems or combination therapies may help mitigate these concerns. In summary, our findings support the potential of CDK1 inhibition as a novel strategy to overcome chemoresistance in PDAC. However, careful consideration of pharmacokinetics, toxicity profiles, dosing regimens, and patient selection will be essential in advancing the CDK1 inhibitor toward clinical application.

One limitation of our study is the lack of validation in the Kras^G12D^-driven murine model of PDAC. Further preclinical studies using in vivo animal models and clinical studies are needed to evaluate the therapeutic potential of targeting this axis. Additionally, while our findings primarily focus on PDAC, it will be important to investigate whether the CDK1–USP33–SIN1 axis has broader implications in other aggressive cancers characterized by high USP33 and SIN1 expressions. In conclusion, our study provides compelling preclinical evidence that targeting the CDK1–USP33 axis may effectively destabilize SIN1, thereby inhibiting tumor growth and overcoming gemcitabine resistance in PDAC. These findings may pave the way for new drug discovery and therapeutic strategies in PDAC treatment.

## Supplementary information

Supplementary Figure

Original data

## Data Availability

All data generated or analyzed during this study are available within the article and Supplementary Files, or available from the authors upon request.
