# Supplementary material for: Phosphorylation of USP33 by CDK1 stabilizes the mTORC2 component SIN1
Source: Cell Death Dis. 2025 Jul 22;16(1):543. doi: 10.1038/s41419-025-07869-6 (PMC12284064; doi:10.1038/s41419-025-07869-6)
Supplement: Supplementary file 1 — Supplementary Figure [file 41419_2025_7869_MOESM1_ESM.pdf]

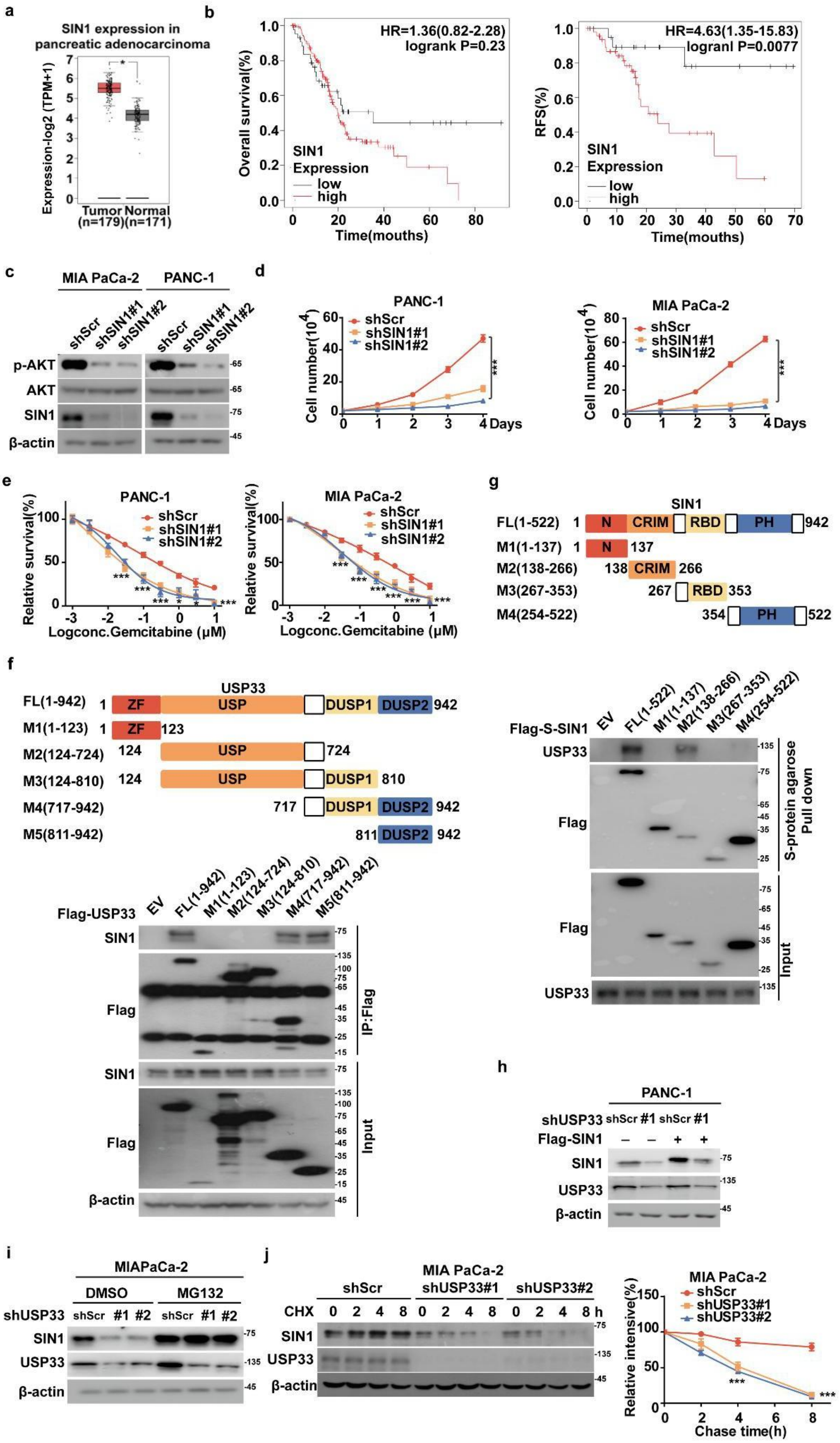
a
SIN1 expression in pancreatic adenocarcinoma
Expression-log2 (TPM+1)
Tumor (n=179)
Normal (n=171)
b
HR=1.36(0.82-2.28)
logrank P=0.23
Overall survival(%)
SIN1 Expression
low
high
Time(mouths)
HR=4.63(1.35-15.83)
logranl P=0.0077
RFS(%)
SIN1 Expression
low
high
Time(mouths)
c
MIA PaCa-2
PANC-1
shScr
shSIN1#1
shSIN1#2
p-AKT
AKT
SIN1
β-actin
d
PANC-1
MIA PaCa-2
shScr
shSIN1#1
shSIN1#2
Cell number(10^4)
Days
e
PANC-1
MIA PaCa-2
Relative survival(%)
Logconc.Gemcitabine (μM)
f
USP33
FL(1-942)
M1(1-123)
M2(124-724)
M3(124-810)
M4(717-942)
M5(811-942)
ZF
USP
DUSP1
DUSP2
Flag-USP33
EV
SIN1
Flag
β-actin
IP:Flag
Input
g
SIN1
FL(1-522)
M1(1-137)
M2(138-266)
M3(267-353)
M4(254-522)
N
CRIM
RBD
PH
Flag-S-SIN1
USP33
Flag
S-protein agarose Pull down
Input
h
PANC-1
shUSP33
Flag-SIN1
SIN1
USP33
β-actin
i
MIAPaCa-2
DMSO
MG132
shUSP33
SIN1
USP33
β-actin
j
MIA PaCa-2
shScr
shUSP33#1
shUSP33#2
CHX
SIN1
USP33
β-actin
Relative intensive(%)
Chase time(h)

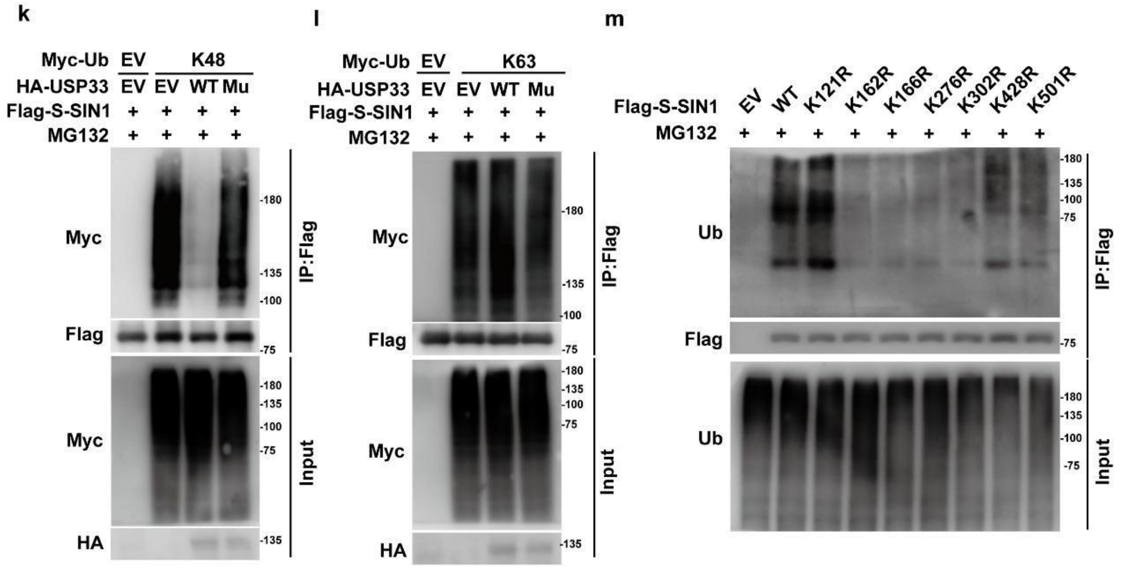


**Supplementary Fig. 1. Identification of USP33 as the Bona Fide Deubiquitinase of SIN1**

(**a**) SIN1 expression in pancreatic adenocarcinoma was obtained in gepia.cancer-pku database. (**b**) Kaplan–Meier survival curves for SIN1 in PDAC were generated using the KM Plotter database (http://kmplot.com). (**c**) MIA PaCa-2 and PANC-1 cells stably expressing shScramble (shScr), shSIN1#1, shSIN1#2 were generated and the protein level of SIN1 was measured by western blot. (**d**) Cell proliferation of cells as in (c) was performed. Data shown as mean ± SD (n=3). (**e**) Cells as in (c) were treated with indicated concentrations of gemcitabine and cell survival was determined. Data shown as mean ± SD (n=4). (**f**) Schematic showing the generation of the USP33 (full-length (FL)), M1 (1-123aa), M2 (124-724aa), M3(124-810aa), M4(717-942aa) and M5 (810-942aa) constructs. ZF, zinc finger; UCH, ubiquitin carboxyl- terminal hydrolase. Full length Flag-USP33 or its truncation mutants were expressed in cells. Cell lysates were subjected to immunoprecipitation with anti-Flag affinity gel. The immunoprecipitates were blotted with indicated antibodies. (**g**) Schematic showing the generation of the SIN1 (full-length (FL), M1 (1-127aa), M2 (138-266aa), M3 (267-353aa), and M4 (354-522aa) constructs. N, N-terminal domain; CRIM, conserved

region in the middle domain; RBD, Ras-binding domain; PH, pleckstrin homology domain. Full length Flag-SIN1 or its truncation mutants were expressed in cells. Cell lysates were pull-downed by S-protein agarose. The immunoprecipitates were blotted with indicated antibodies. (**h**) Control (shScramble) or USP33-depleted PANC-1 cells were transfected with Empty Vector (EV) or Flag-SIN1. The protein levels of SIN1 and USP33 were examined by western blot. (**i**) PANC-1 cells stably expressing shScramble (shScr), shUAP33#1, shUSP33#2 were treated with DMSO or MG132 (10 μM) for 10 h. Western blot was performed. (**j**) Cycloheximide pulse-chase assay was performed in PANC-1 cells stably expressing shScramble(shScr), shUAP33#1, shUSP33#2. SIN1 protein levels relative to β-actin was measured by image J. Data shown as mean ± SD (n=3). (**k-l**) Cells stably expressing Empty Vector (EV), HA-USP33 WT or C194S H673Q (Mu) mutant were transfected with indicated plasmids and treated with MG132 for 10 h. Cell lysates were subjected to immunoprecipitation with anti-Flag affinity gel and the ubiquitination of SIN1 protein was examined by western blot. (**m**) Cells were transfeced with empty vector, Flag-S-SIN1 WT or different KR mutants and then treated with MG132 for 10 h. SIN1 was immunoprecipitated with anti-Flag affinity gel and the polyubiquitylated SIN1 protein was examined by western blot. Data were analyzed by two-sided one-way ANOVA in (**d, e, j**). Statistic significance was determined using the following p-values: * $P < 0.05$; ** $P < 0.01$; *** $P < 0.001$.

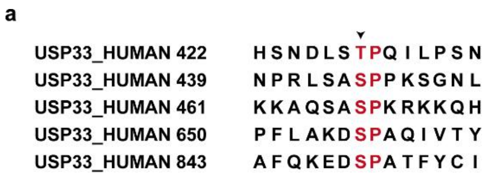


**Supplementary Fig. 2. CDK1 binds and phosphorylates USP33.**

(**a**) Candidate sites of USP33 which conforming to CDK substrate consensus. Arrows, serine or threonine residues that could be phosphorylated by CDK.

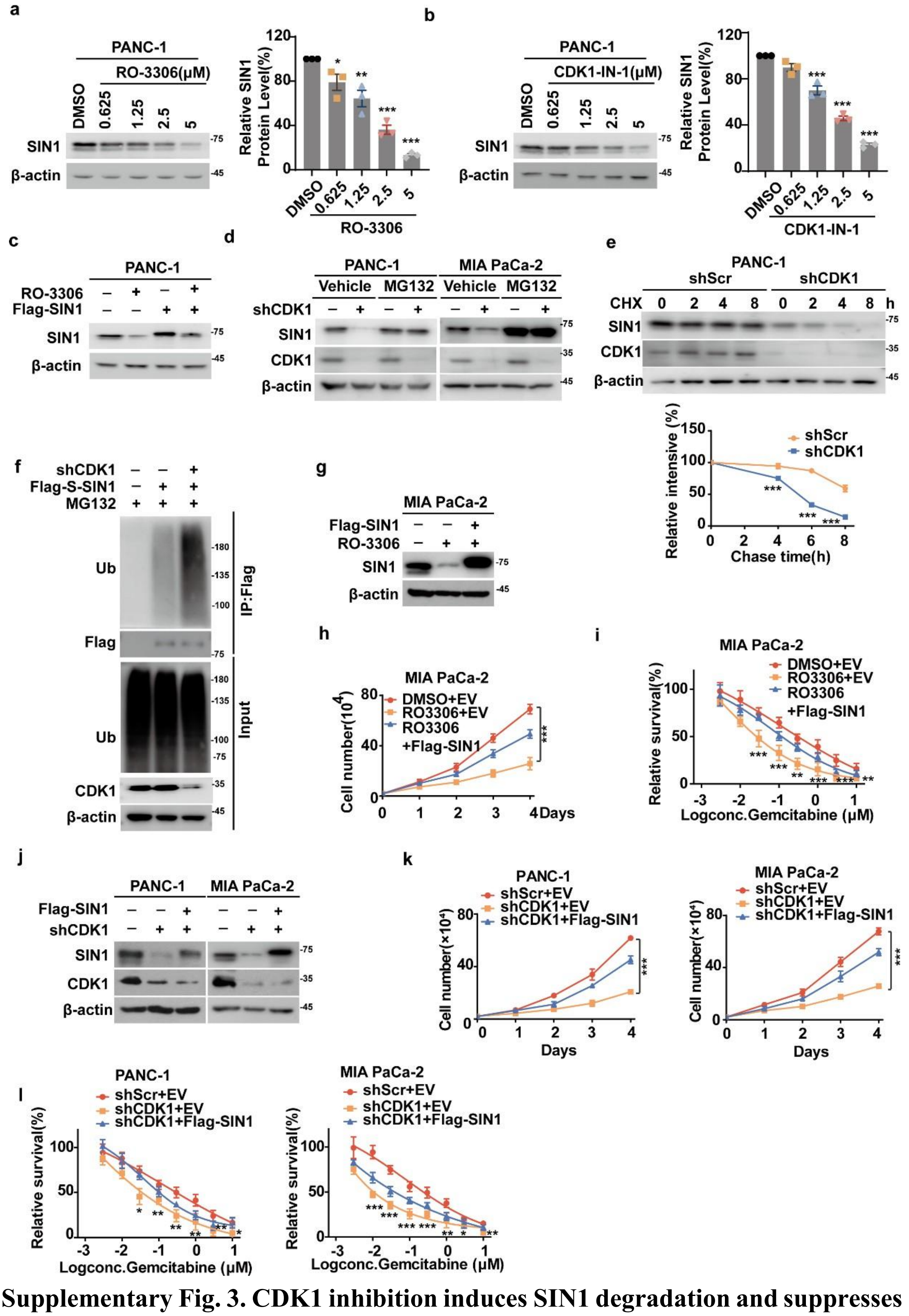


**Supplementary Fig. 3. CDK1 inhibition induces SIN1 degradation and suppresses PDAC progression**

(**a-b**) PANC-1 cells were treated with DMSO or indicated concentrations of RO3306 (**a**) or CDK1-IN-1 (**b**) for 24 h and western blot was performed with indicated

antibodies. The relative level of SIN1 to β-actin was measured by image J. Data shown as mean ± SD (n=3). (**c**) PANC-1 cells were transfected with Empty Vector (EV) or Flag-SIN1, and then treated with either DMSO or RO-3306 (5 μM) for 24 h. And protein levels of SIN1 were examined by western blot. (**d**) PANC-1 and MIA PaCa-2 cells stably expressing shScramble (shScr), shCDK1 were treated with DMSO or MG132 and western blot was performed with indicated antibodies. (**e**) Cycloheximide pulse-chase assay was performed in PANC-1 cells; The relative level of SIN1 to β-actin was measured by image J. Data shown as mean ± SD (n=3). (**f**) PANC-1 cells treated with DMSO or RO-3306 (5 μM) for 24 h were transfected with indicated plasmids and treated with MG132 for 10 h. Cell lysates were subjected to immunoprecipitation with anti-Flag affinity gel and the ubiquitination of SIN1 protein was examined by western blot. (**g**) MIA PaCa-2 cells pretreated with DMSO, RO-3306 (5 μM) or RO-3306 transfected with Flag-SIN1 were generated and western blot was performed with indicated antibodies. (**h**) Cell proliferation assay was performed in PANC-1 cells as in (**g**). Data shown as mean ± SD (n=3). (**i**) Cells as in (g) were treated with indicated concentrations of gemcitabine and cell survival was determined. Data shown as mean ± SD (n=4). (**j**) PANC-1 and MIA PaCa-2 cells stably expressing shScramble (shScr), shCDK1 were transfected with empty vector or Flag-SIN1 and western blot was performed with indicated antibodies. (**k**) Cell proliferation assay was performed in PANC-1 and MIA PaCa-2 cells as in (j). Data shown as mean ± SD (n=3). (**i**) Cells as in (j) were treated with indicated concentrations of gemcitabine and cell survival was determined. Data shown as mean ± SD (n=4). Data were analyzed by two-sided Student's *t* test in (**e**), by two-sided one-way ANOVA in (**a, b, h, i, k, l**). Statistic significance was determined using the following p-values: * $P < 0.05$; ** $P < 0.01$; *** $P < 0.001$.

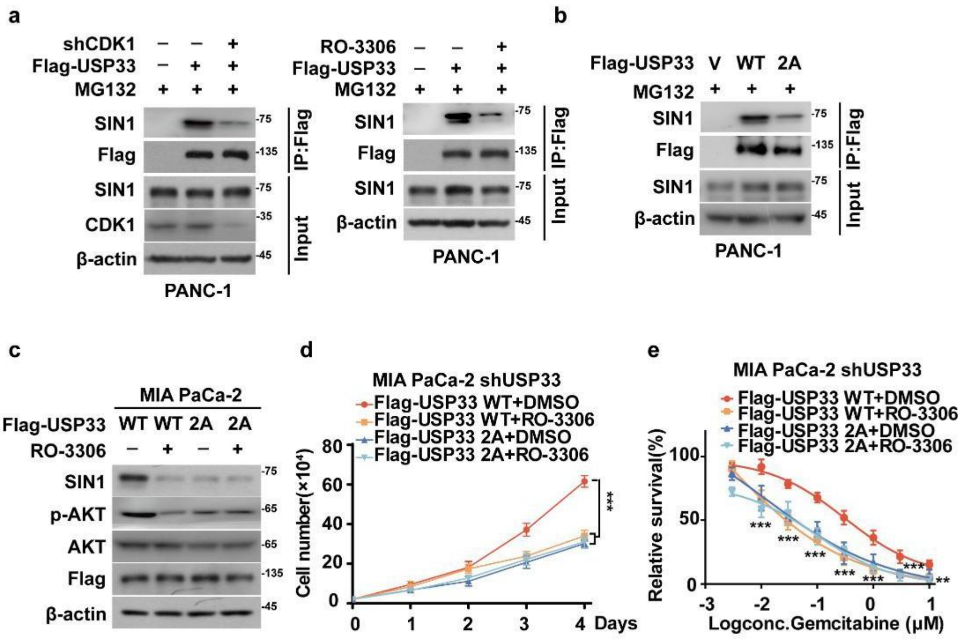


**Supplementary Fig. 4. CDK1-mediated phosphorylation of USP33 is pivotal for SIN1 stability and PDAC progression**

(**a**) PANC-1 cells stably expressing shScramble (shScr) or shCDK1 were generated and transfected with empty vector or Flag-USP33. Cell lysates were subjected to immunoprecipitation with anti-Flag affinity gel, and western blot was performed with indicated antibodies (left). Cells were transfected with vector or Flag-USP33 and then treated with DMSO or RO-3306 (5 μM). Cell lysates were immunoprecipitated with anti-Flag affinity gel, and western blot was performed with indicated antibodies (right). (**b**) PANC-1 cells stably expressing empty vector (EV), Flag-USP33 WT or 2A and treated with MG132 (10 μM). Cell lysates were subjected to immunoprecipitation with anti-Flag affinity gel and western blot was performed with indicated antibodies. (**c**) MIA PaCa-2 cells with endogenous USP33-deficiency were transfected with indicated plasmids and western blot was performed. (**d**) Cell proliferation assay was performed in MIA PaCa-2 cells as in (c). Data shown as mean ± SD (n=3). (**e**) Cells as in (c) were treated with indicated concentrations of gemcitabine and cell survival was determined. Data shown as mean ± SD (n=4). Data were analyzed by two-sided one-way ANOVA in (**d, e**). Statistic significance was determined using the following p-values: * *P < 0.05;* ** *P < 0.01;* *** *P < 0.001.*
