# Supplementary material for: Phosphorylation of USP33 by CDK1 stabilizes the mTORC2 component SIN1
Source: Cell Death Dis. 2025 Jul 22;16(1):543. doi: 10.1038/s41419-025-07869-6 (PMC12284064; doi:10.1038/s41419-025-07869-6)

Fig.1c

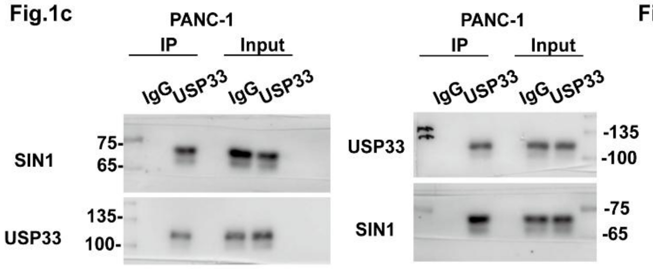

Fig.1d

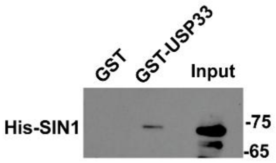

Fig.1e

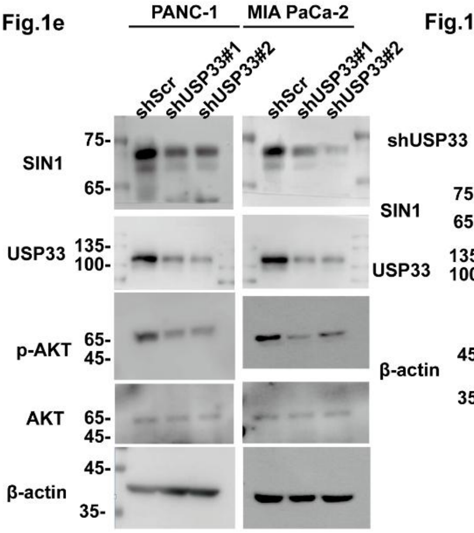

Fig.1g

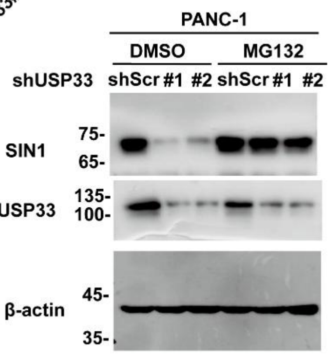

Fig.1h

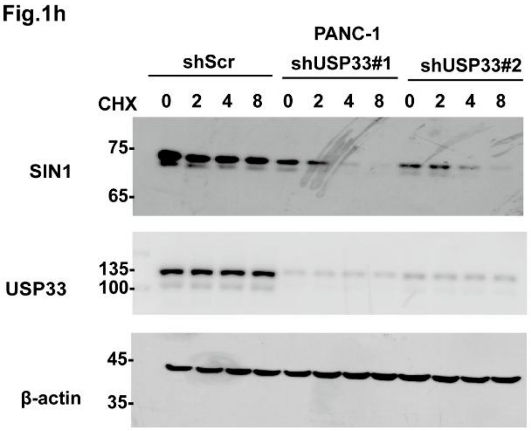

Fig.1i

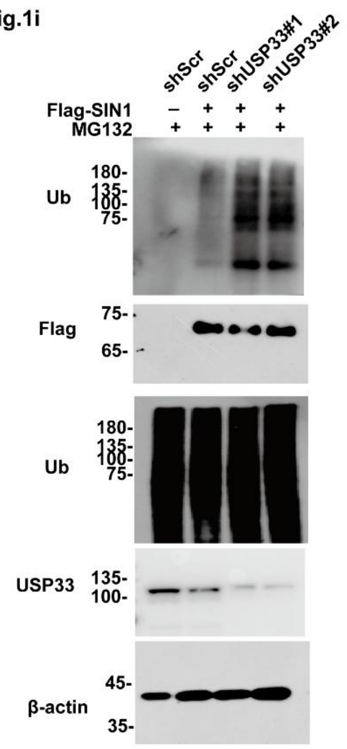

Fig.1j

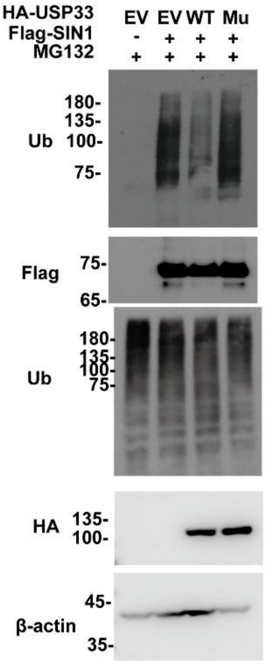

Fig.1k

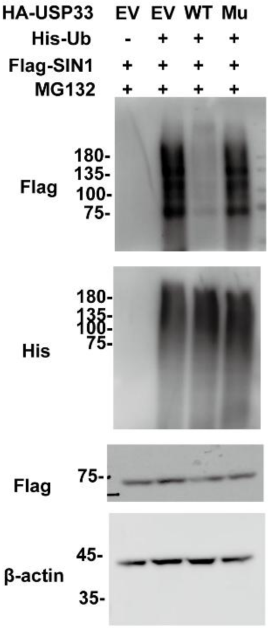

Fig.1l

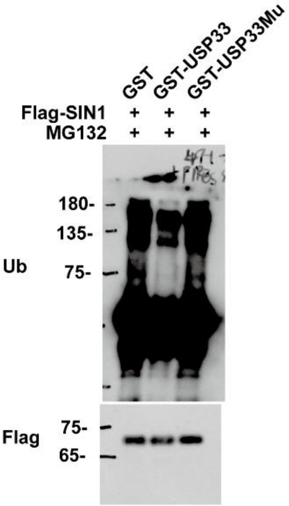

Fig.1m

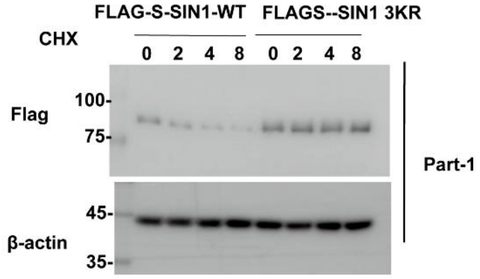

Fig.1n

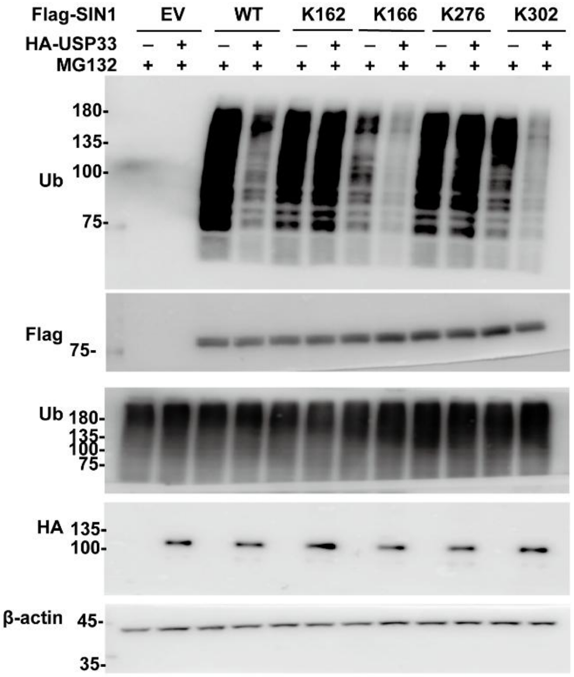

Supplementary Fig.1c

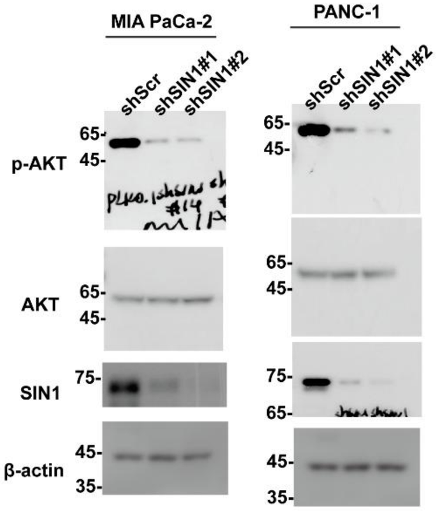

Supplementary Fig.1f

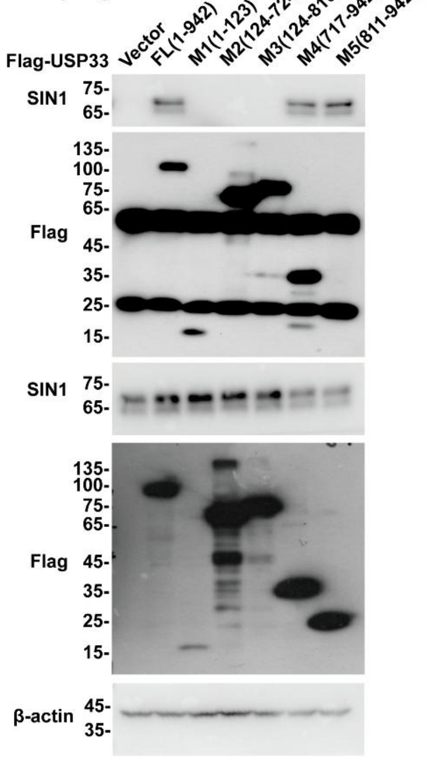

Supplementary Fig.1g

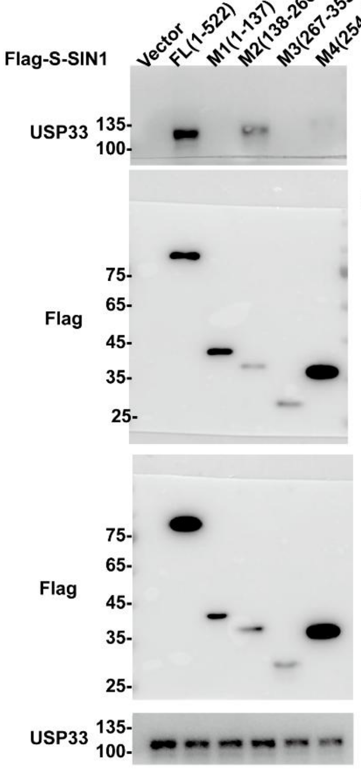

Supplementary Fig.1h

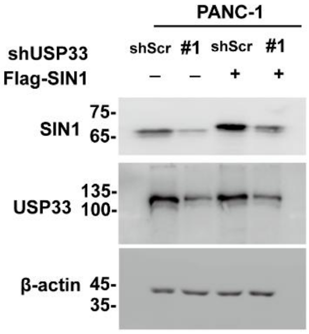

Supplementary Fig.1i

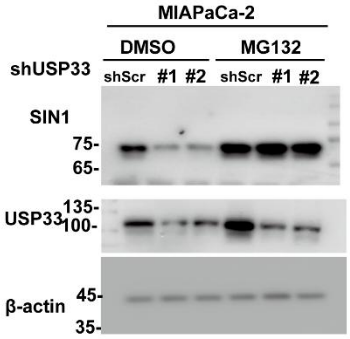

Supplementary Fig.1j

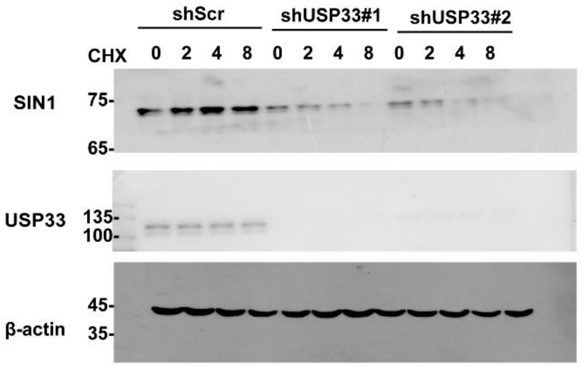

Supplementary Fig.1k

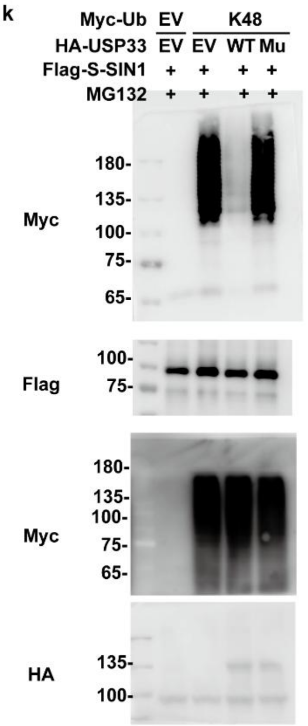

Supplementary Fig.1l

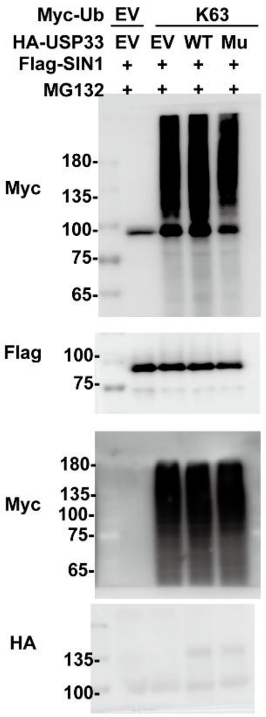

Supplementary Fig.1m

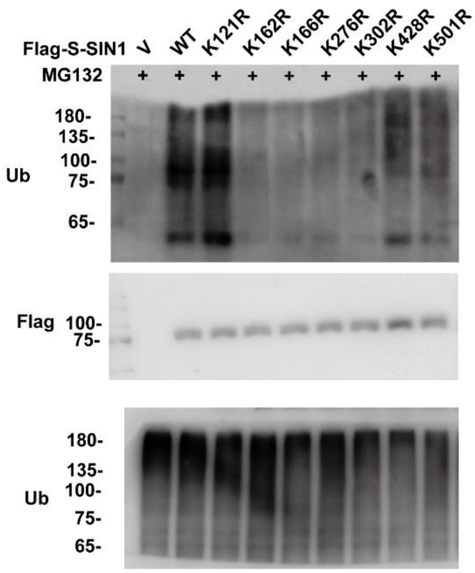

Fig.2a

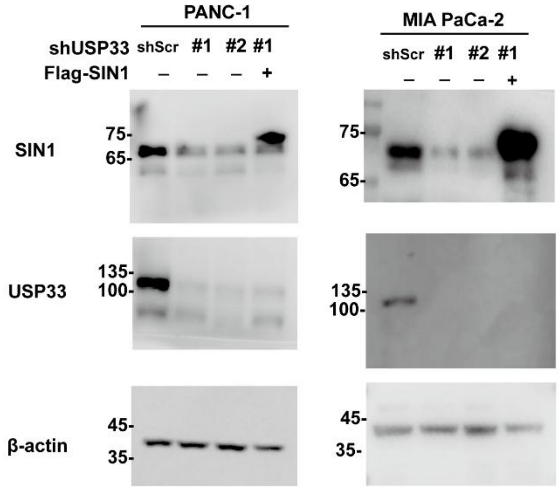

Fig.2i

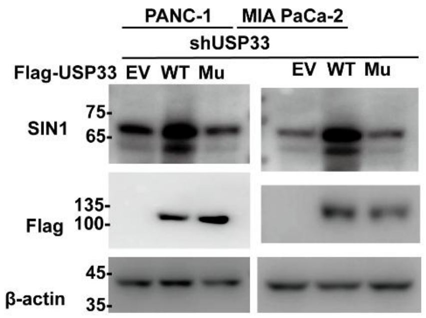

Fig.3b

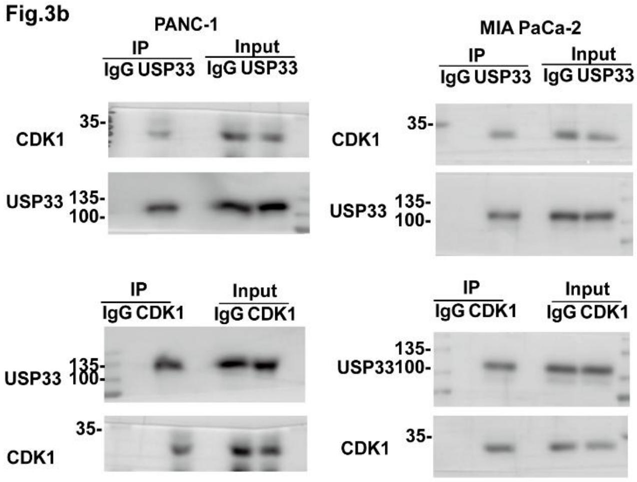

Fig.3c

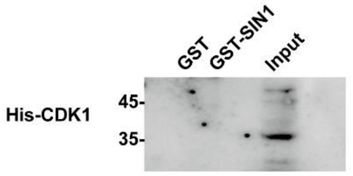

Fig.3d

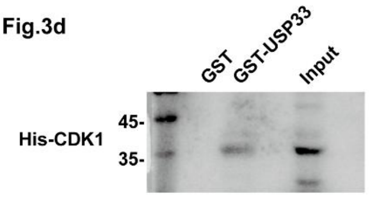

Fig.3e

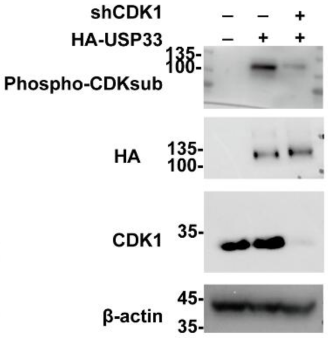

Fig.3f

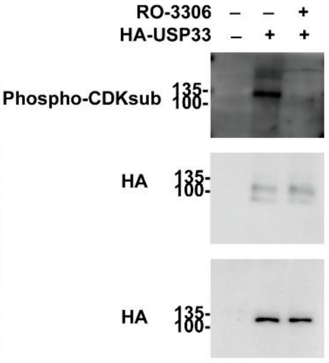

Fig.3g

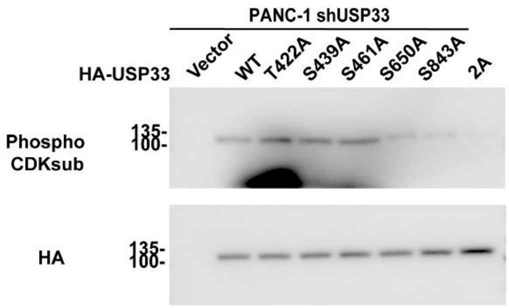

Fig.3h

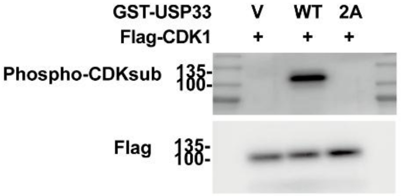

Fig.4a

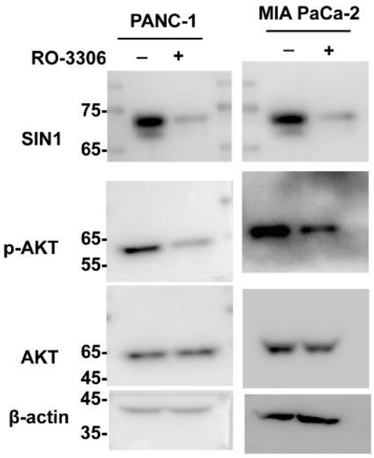

Fig.4c

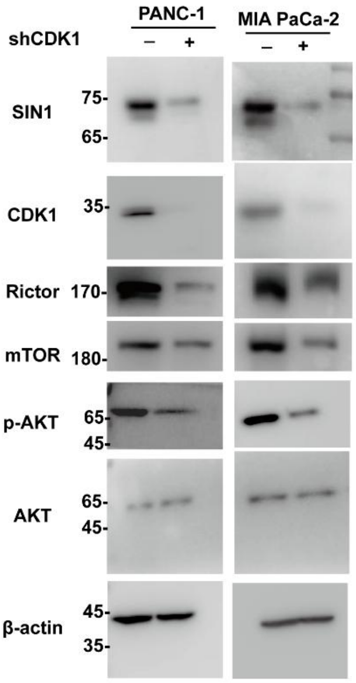

Fig.4e

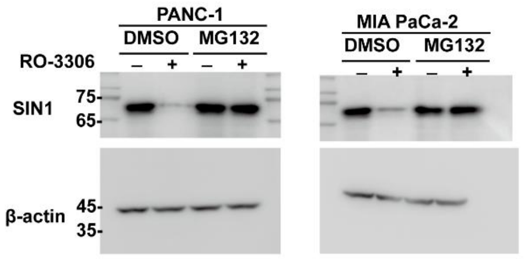

Fig.4f

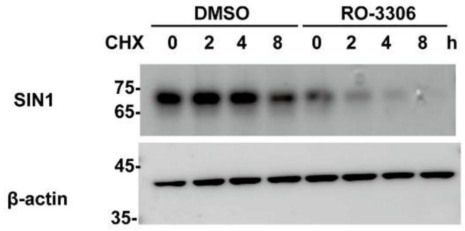

Fig.4g

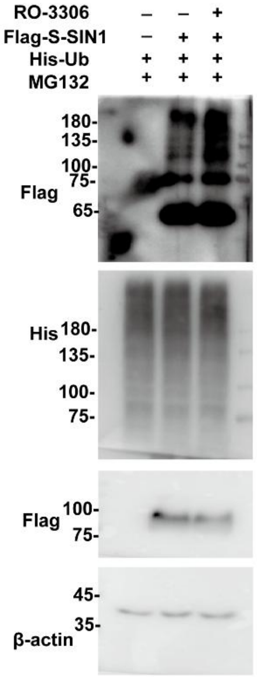

Fig.4h

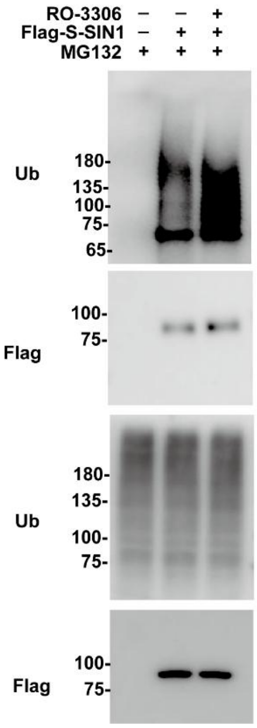

Fig.4i

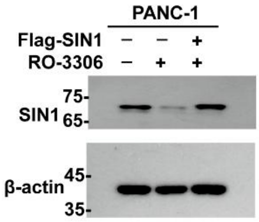

Supplementary Fig.3a

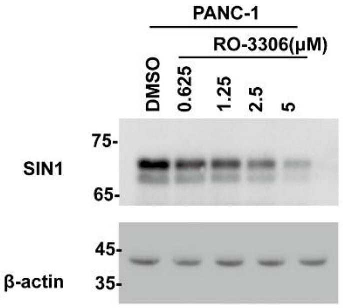

Supplementary Fig.3b

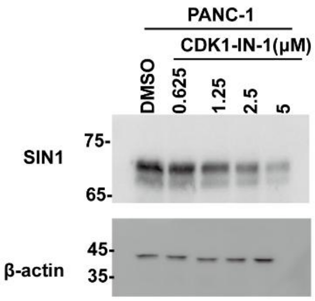

Supplementary Fig.3c

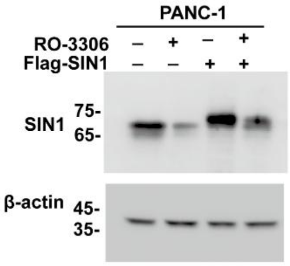

Supplementary Fig.3d

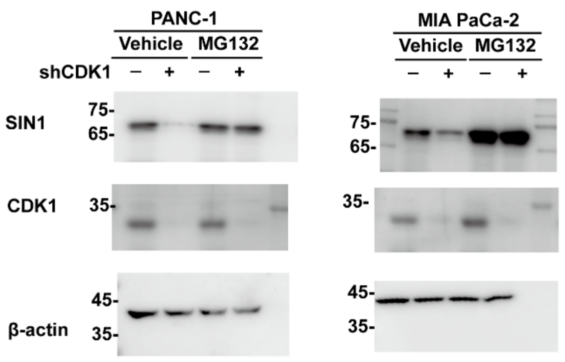

Supplementary Fig.3e

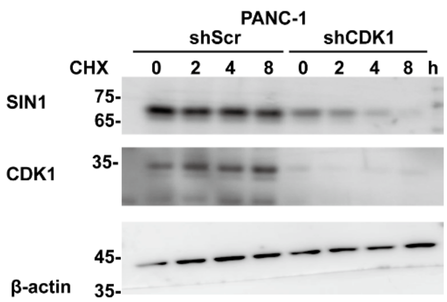

Supplementary Fig.3f

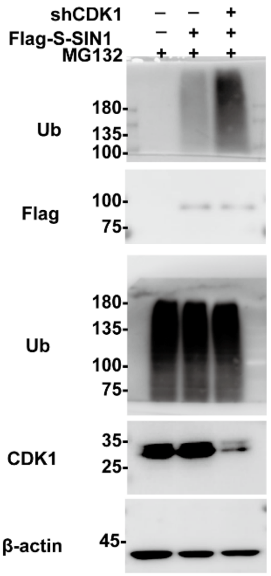

Supplementary Fig.3g

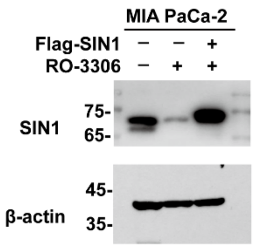

Supplementary Fig.3j

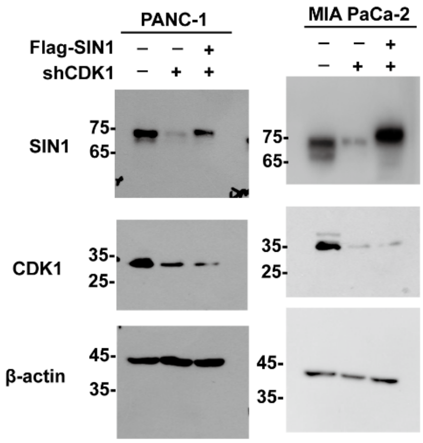

Fig.5a

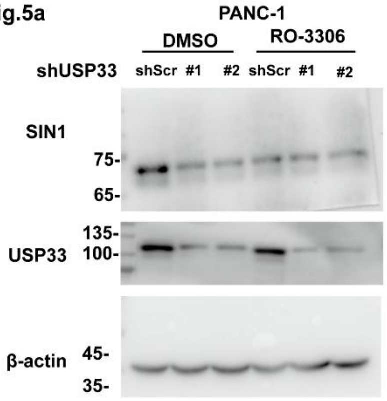

Fig.5b

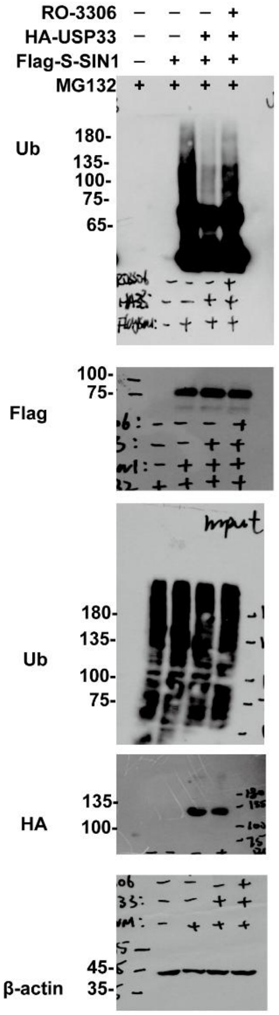

Fig.5c

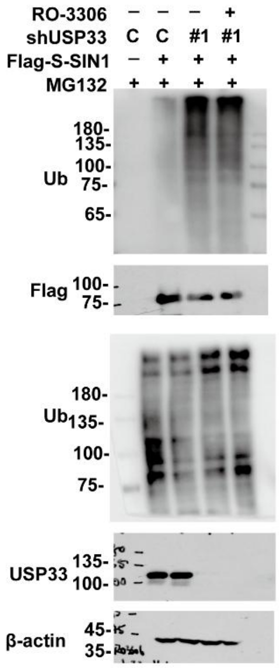

Fig.5d

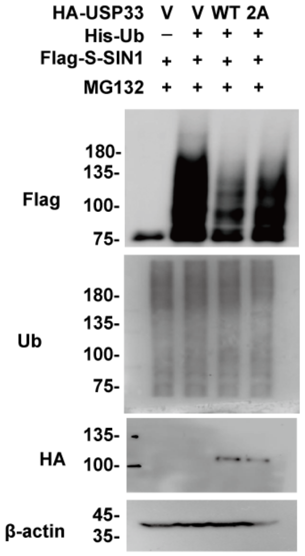

Fig.5f

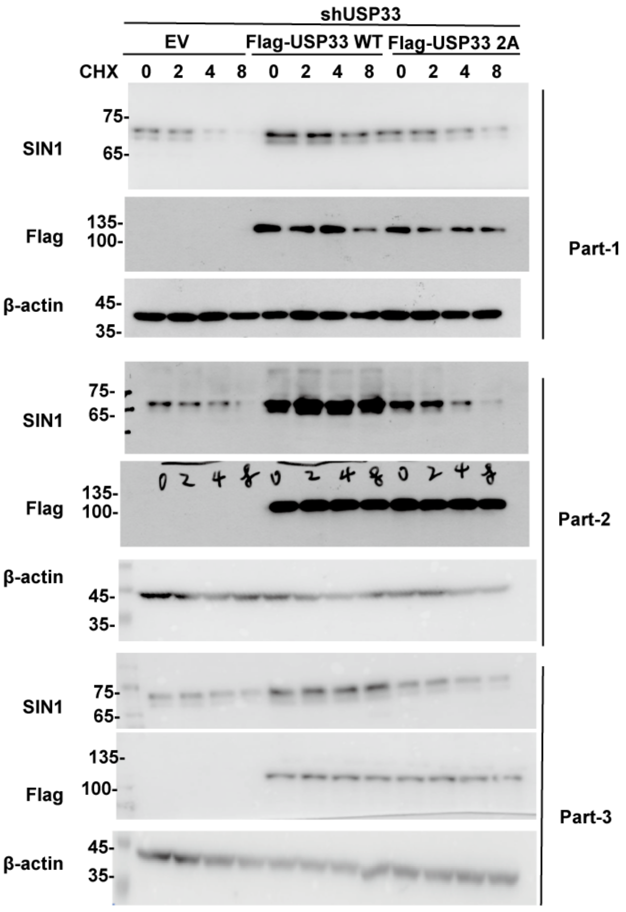

Fig.5e

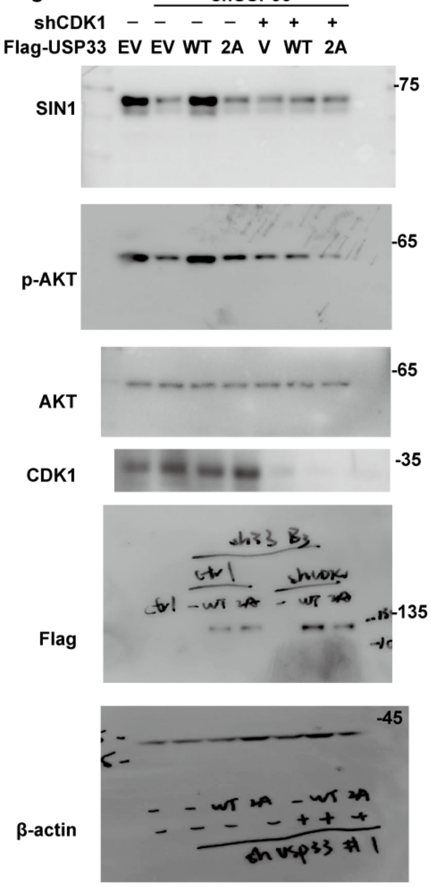

Fig.5g

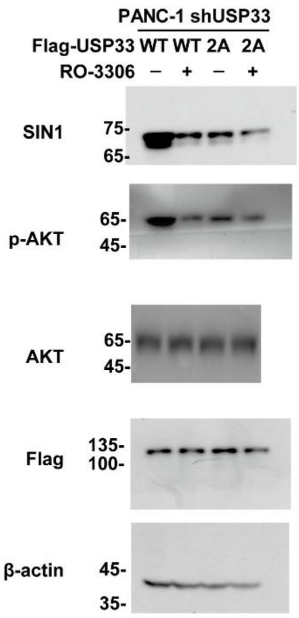

Supplementary Fig.4a

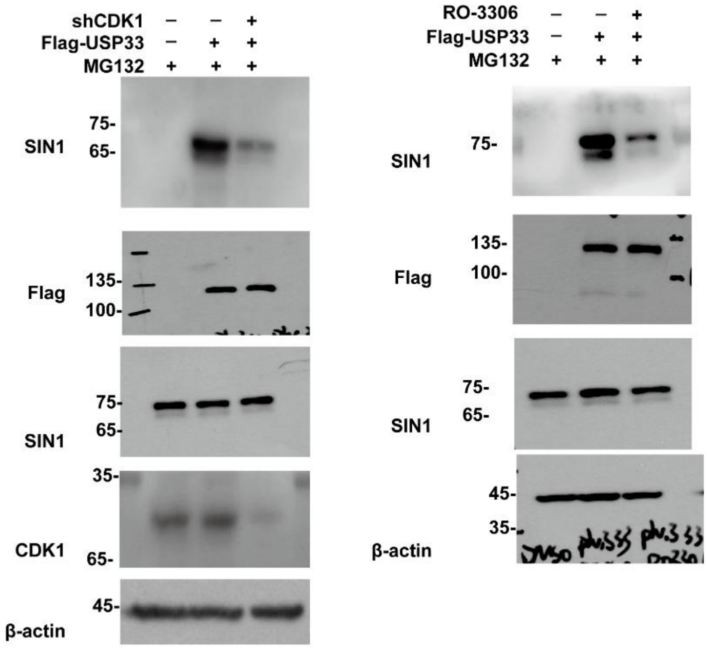

Supplementary Fig.4b

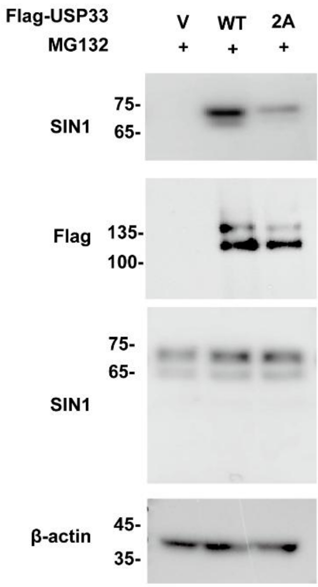

Supplementary Fig.4c

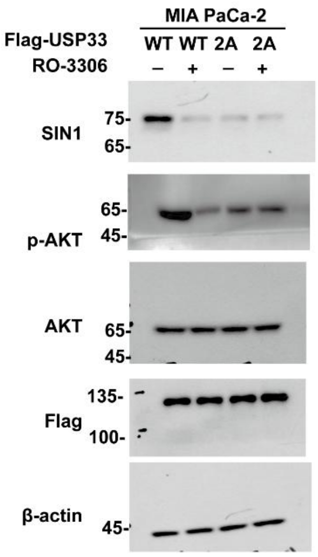

Supplement: Supplementary file 2 — Original data [file 41419_2025_7869_MOESM2_ESM.pdf]
